# New Paradigm in NKT Cell Antigens: MCS‐0208 (2‐(Hydroxymethyl)phenylthio‐phytoceramide) – an Aryl‐Phytoceramide Compound with a Single Hydroxyl Group Stimulates NKT Cells

**DOI:** 10.1002/cmdc.202000992

**Published:** 2021-06-17

**Authors:** Roser Borràs‐Tudurí, Anna Alcaide, Sandrine Aspeslag, Lorena Usero, Carmen Serra, Carme Roura‐Mir, Dirk Elewaut, Amadeu Llebaria

**Affiliations:** ^1^ Medicinal Chemistry and Synthesis (MCS) Laboratory Institut de Química Avançada de Catalunya (IQAC) Consejo Superior de Investigaciones Científicas (CSIC) C/ Jordi Girona 18–26 08034 Barcelona Spain; ^2^ Unit for Molecular Immunology and Inflammation VIB-Center for Inflammation Research Ghent Belgium; ^3^ Department of Internal Medicine and Pediatrics Ghent University Ghent Belgium; ^4^ Laboratori d'Immunologia Cel⋅lular Institut de Biotecnologia i Biomedicina Universitat Autònoma de Barcelona Bellaterra 08193 Barcelona Spain

**Keywords:** NKT cells, alfa-galactosylceramide, aryl-ceramide, immunostimulation

## Abstract

Natural Killer T (NKT) cells play an important role in the immune response and can be activated by glycolipids presented by CD1d protein. We present MCS‐0208, an unprecedented arylthioether‐phytoceramide able to induce potent invariant NKT (iNKT) cell activation, notably when tested in human iNKT cells. This arylsphingolipid analog has a simple phenyl group containing a single hydroxyl substituent as a surrogate of the sugar ring. The phenylthioether structure contrasts with α‐galactosylceramide (**1**), the prototypical glycolipid used to induce iNKT cell stimulation, where the galactose 2’‐OH and 3’‐OH substituents are accepted as the minimal footprint and considered critical for NKT T cell receptor (TCR) recognition. A computational study supports the recognition of aromatic compound by the CD1d and TCR proteins as radically new structures for NKT cell stimulation.

## Graphical abstract

**Stimulating NKT cells**. Breaking the rules of chemical structure of antigens for NKT cells stimulation, a single phenyl‐hydorxymethyl moiety is able to stimulate NKT cells via CD1d presentation. The replacement of alpha‐galacose by hydroxymethyl‐phenyl‐thioether lead to a interesting new NKT cell antigen, opening an unexplored chemical space for new antigens development with promising profile.

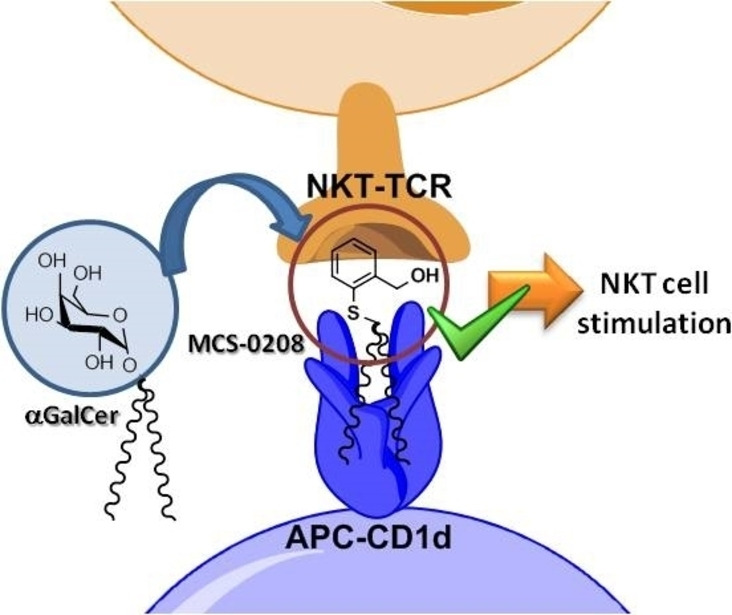

During the past two decades, extensive research was done on designing, synthesizing and evaluating new α‐galactosylceramide (αGalCer) derivatives.^[1]^ This galactose α‐linked to a phytoceramide lipid was found during a screening of anticancer agents of marine sponge extractions showing the induction of promising, potent and antitumor response.^[2]^ After this finding, intensive efforts were directed to decipher the mechanism of action of this compound resulting in the identification of Natural Killer T (NKT) cells, a new subtype of lymphocytes with Natural Killer (NK) cells and T lymphocytes characteristics. Another important finding was that the particular T cell receptor was restricted to CD1d‐antigen complex. CD1d is a non‐classical MHC‐class I that recognizes and presents lipid antigens and in particular glycosphigolipid derivatives, instead of peptides. The prototypical NKT cell stimulator αGalCer, displays a very potent activity and induces both Th1 and Th2 opposite responses from NKT cells with several undesired effects. Another undesirable effect of αGalCer was its overstimulation leading to an unresponsiveness state of the cells. Therefore, plenty of studies were conducted to modify the structure of αGalCer in order to optimize its physicochemical and biological properties. Hundreds of new compounds were synthesized and biologically studied concluding which part of the αGalCer structure has an impact on the strength or the type of response (Figure 1). In general, most chemical efforts were focused on the galactose moiety and the lipid chains. The sugar was extensively studied exploring both α‐ and β‐linked glycolipids, the configuration of the hydroxylated carbons (introducing glucose, mannose or other sugars) and hydroxyl substitution or fuctionalization. There is a general agreement on the critical importance of 2’‐OH and 3’‐OH for CD1d recognition while the modification on other parts of the galactose can be somehow modified to modulate the interaction with the T cell receptor (TCR) and, as a result, modulate the NKT cell response. The other broadly studied part of the αGalCer was the lipid chains, exploring in both tails the length impact,[^3^, ^4^] ramification or substitution, and the introduction of other groups such as aromatic rings,[^5^, ^6^, ^7^, ^8^] polar groups^[9]^ or halogens. Recently it was published a new derivative able to bind covalently to the CD1d antigen binding groove, increasing ligand affinity^[10]^ and inducing a Th2 polarizing activity.

**Figure 1:**
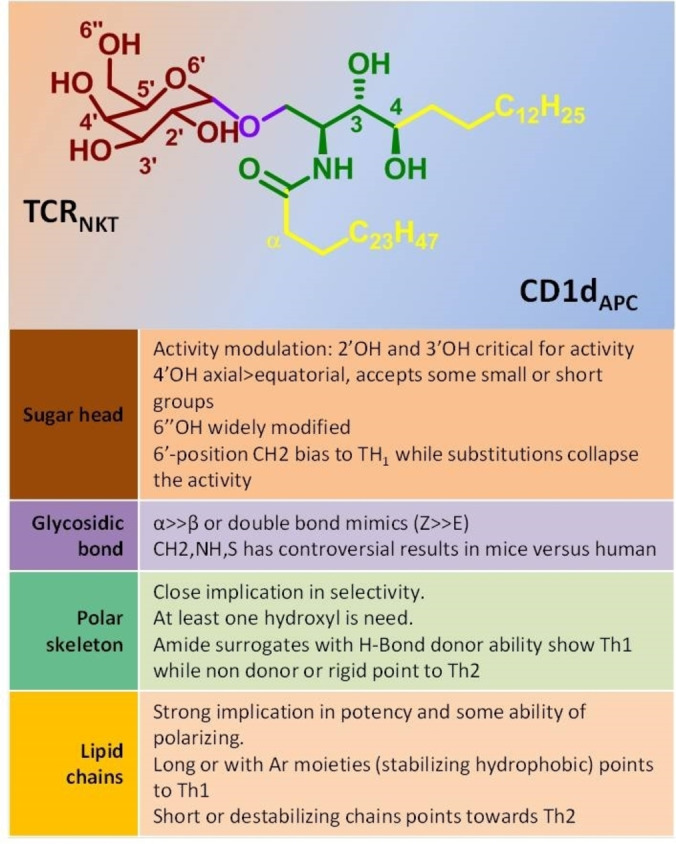
General overview of αGalCer chemical structure and the impact of each part in the biological response modulation.

Our group studied non‐glycosidic compounds such as aminocyclitol‐ceramide derivatives HS44 and HS161, (compounds **3** and **4** in Figure 2), showing high levels of cytokine prodution and anticancer *in vivo* activity.[^11^, ^12^]

**Figure 2:**
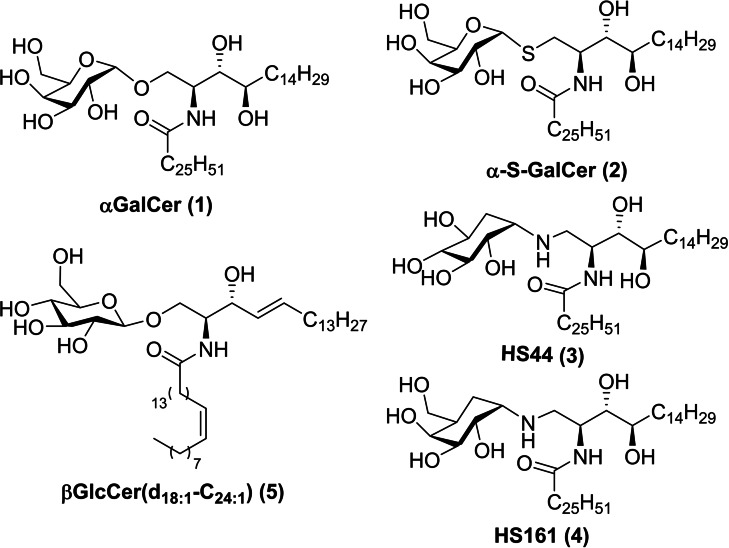
Chemical structure of some aGalCer derivatives.

Taking advantage of our group background on the chemical synthesis of a great diversity of sphingosine and phytospgingosine derivatives in several fields from inhibitors of sphingolipid‐metabolizing enzymes^[13]^ to NKT cell modulators,[^11^, ^12^, ^14^] some new thiosugar derivatives from our chemical collection were screened as candidates for NKT cell modulation (compounds **6**,^[13]^ **7** ^[13]^ and **8** ^[13]^ in Figure 3). These compounds presented a chemical structure related to or similar to β‐glucosylceramide **5** (Figure 2), which was postulated as endogenous ligand candidate^[15]^ at the time of this work was started however it was finally refused.[^16^, ^17^]

**Figure 3:**
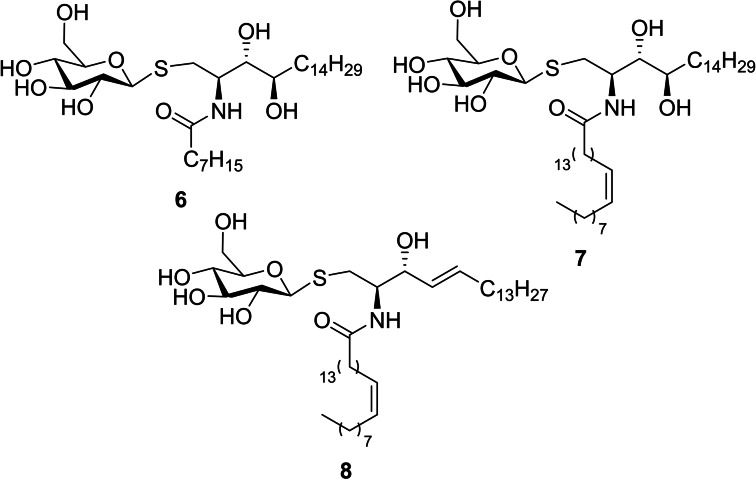
Chemical structure of new β‐S‐Glucosylceramide derivatives.

Immunostimulator potency of these compounds was measured by IL‐2 production of splenocites from Balb‐6 mice *in vitro*. Product **6** and **8** induced low levels of IL‐2 secretion only comparable to that of vehicle (Figure 4). Only compound **7** showed a slightly higher amount of cytokine secretion at the highest dose (1000 ng/mL).

**Figure 4:**
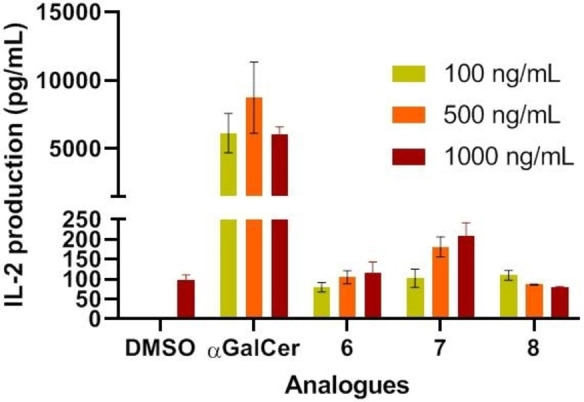
*In vitro* IL‐2 secretion upon NKT cell stimulation with β‐S‐GlcCers. Splenocites were cocultured with each compound in triplicate. Error bars indicate SD values.

Considering that α‐S‐GalCer (**2**), the S‐linked analogue of αGalCer (**1**), was inactive when tested in mice cells,^[18]^ the low levels of cytokine production of our new compounds were not surprising. Moreover the lack of activity in the synthesized β‐thioglucosylceramide analogues **6**, **7** and **8** could not be entirely attributed to the β‐configuration of the sugar moiety and other factors like the length of the *N*‐acyl chain and its degree of saturation, or the fact that these analogues are *S*‐glycosides, should be considered. Taking into account that compound **7** was more active than the O‐linked analogue β‐GlcCer_d18:1‐C24:1_ (**5**),[^16^, ^17^] we decided to explore the NKT activity of our chemical library of non glycosidic ceramide‐derivatives with totally different “polar” heads non‐sugar related such as compounds **9**,^[13]^ **10**,^[13]^ **11** ^[13]^ and **12** ^[19]^ (Figure 5). Moreover, being aware of the importance of the acyl chain length, MCS‐0208 (**13**, Figure 5) was synthesized with the same ceramide skeleton as αGalCer (see Supporting Info for chemical details). To this purpose, initial assays at the highest dose (1000 ng/mL) were performed *in vitro* with splenocites from Balb‐6 mice (Figure 6).

**Figure 5:**
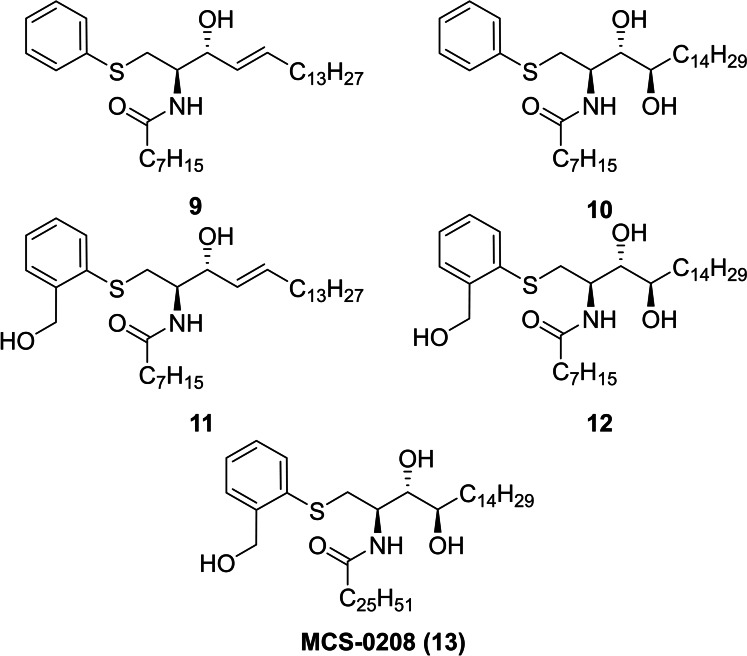
Chemical structure of new ArCer family.

**Figure 6:**
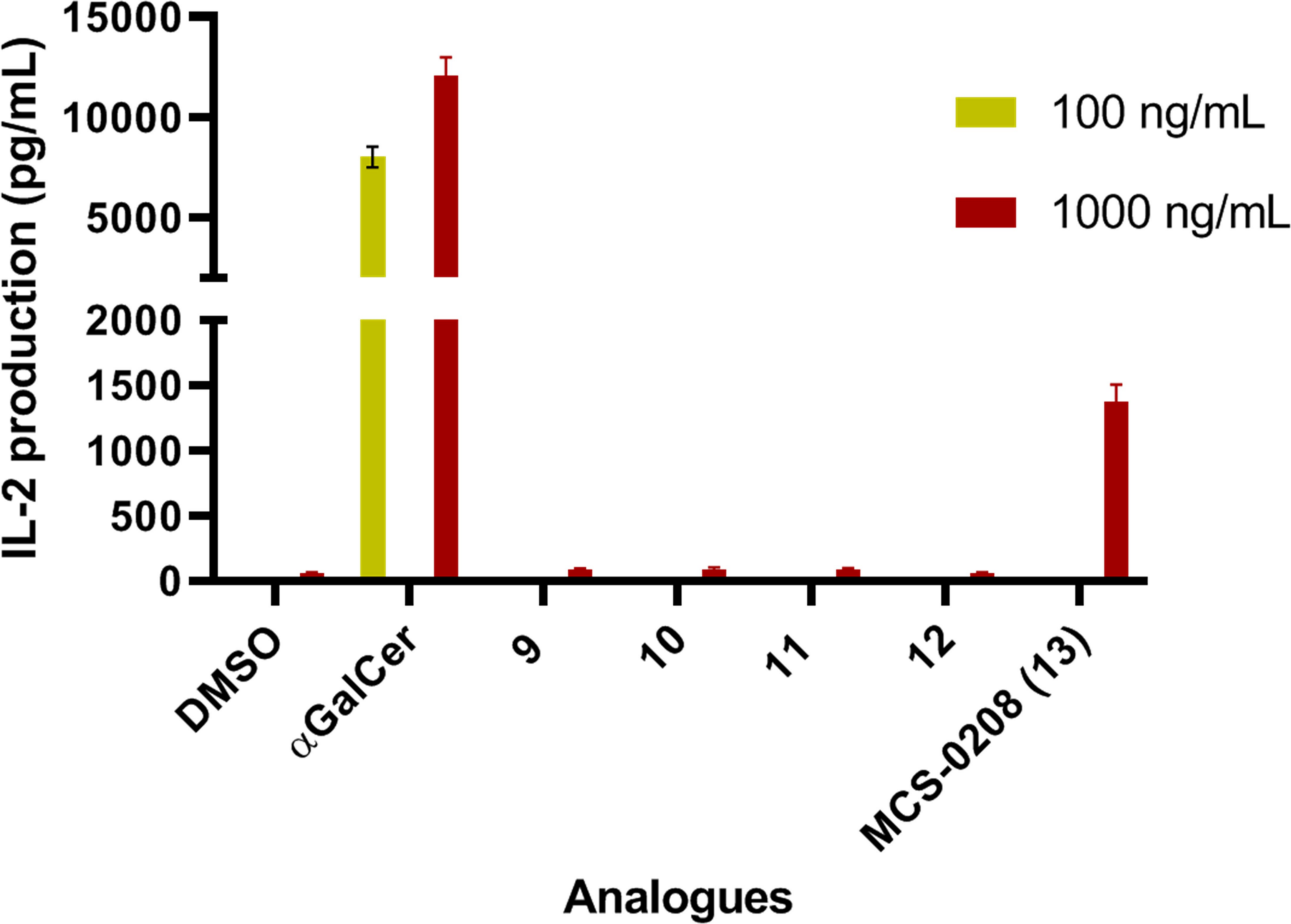
*In vitro* IL‐2 secretion induced by sphingolipid analogues at 1000 ng/mL. Splenocites from BL6 mice were cocultured with each compound in triplicate. Error bars indicate SD values.

The *in vitro* experiments showed that most of the compounds of this set did not activate iNKT responses in a meaningful manner with the only exception of MCS‐0208 (**13**) that showed stronger induction of IL‐2 secretion, but it was not as active as αGalCer, used as reference compound (Figure 6). It is important to highlight the results of this new family of thioaryl‐ceramides (ArCer). The NKT activity of MCS‐0208 (**13**) was rather unexpected because the structure of the group attached at position 1 of the ceramide skeleton is very different from any sugar mimetic. Furthermore, the other analogues with closer structures to MCS‐0208 (**13**), such as compounds **9**, **10**, **11** or **12** induced lower levels of IL‐2 secretion. The main difference was on the *N*‐acyl chain length what was in the established structural effects of the *N*‐acyl groups required for CD1d recognition. In same line of Structure‐Activity studies, the short acyl chain was related to Th2 polarized activity^[2]^ what could explain the lower levels of IL‐2 (a cytokine included among Th1 ones) of compounds **9** to **12**.

In order to compare the potencies of the new ArCer with the β‐*S*‐GlcCer family, we synthesized compound **14** (Figure 7), a β‐*S*‐GlcCer with the same ceramide skeleton than MCS‐0208 (**13**), and they were evaluated together with a dose response of our new hit compound. Interestingly, MCS‐0208 (**13**) showed consistent dose responses, and compound **14** was 2‐fold less active than MCS‐0208 (**13**) at the same dose in spite of the presence of the thiosugar (Figure 7).

**Figure 7:**
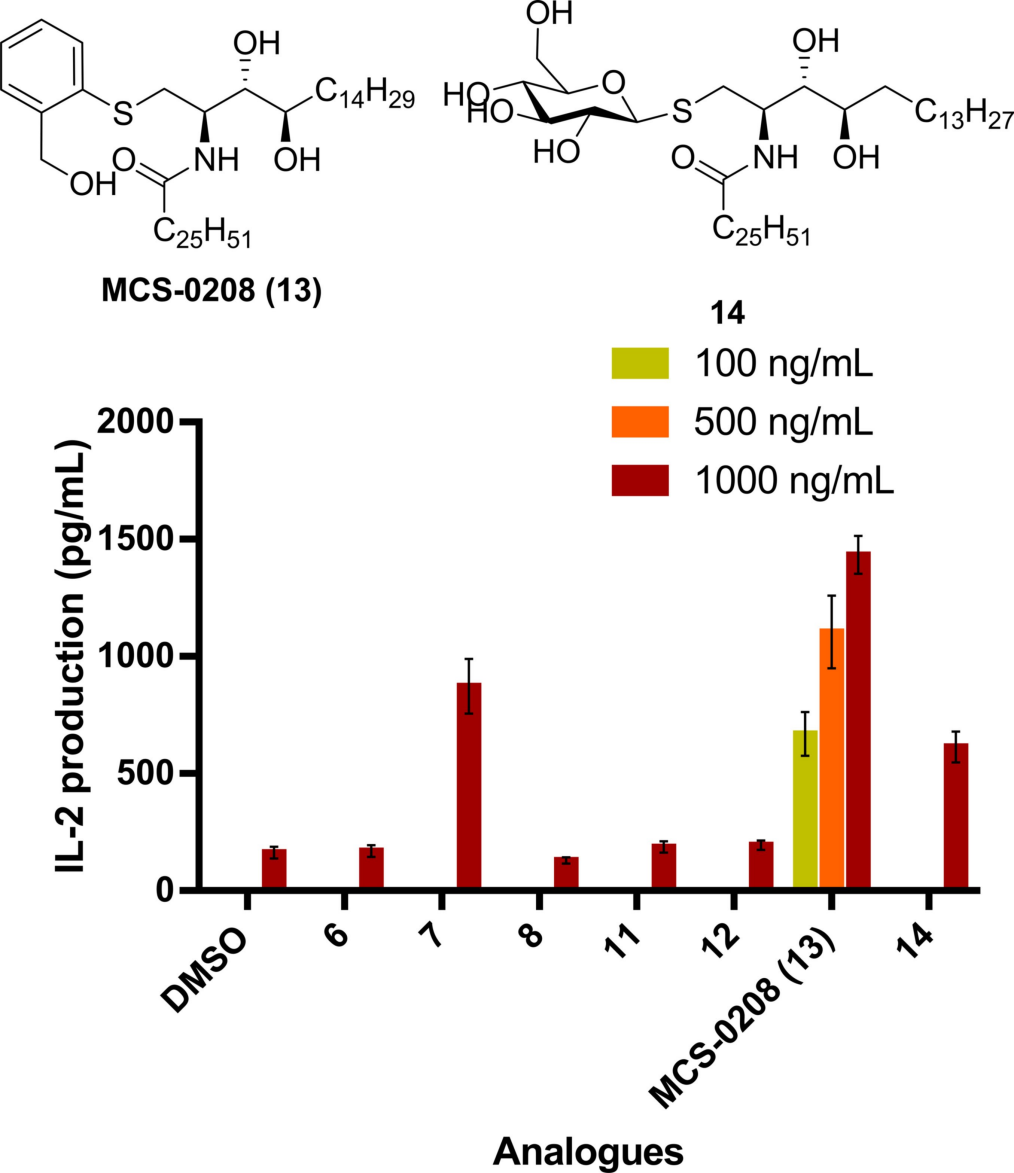
Comparative *in vitro* IL‐2 secretion of ArCers and compound **14**. Splenocites from BL6 mice were cocultured with each analogue in triplicate. Error bars indicate SD values.

In view of these results, it was evident the interest in going further and test the activity of compounds **7**, MCS‐0208 **13** and **14** *in vivo*. Although it was not expected to reach similar αGalCer cytokine production levels, we were more interested in evaluating the activity of the new MCS‐0208 (**13**) compared to other weak agonists with glycosylated ceramide structures. Thus, lipids were tested *in vivo* and were administrated intraperitoneally (5 μg) in PBS to BL6 and 18‐/‐ mice and cytokine secretion was measured on mice sera by ELISA assay (IL‐4 at 4 hours and IFN‐γ at 16 hours) using αGalCer as a positive control. *In vivo* assays confirmed our *in vitro* results showing an agonist profile for all three compounds, but moreover confirmed that MCS‐0208 (**13**) was a stronger agonist than β‐*S*‐GlcCer's **7** and **14** (Figure 8). Interestingly, MCS‐0208 (**13**) was more active than its corresponding thiosugar analogs **7** and **14**, and in all cases a Th1 polarization profile was observed, with higher production of IFN‐γ than IL‐4. In addition MCS‐0208 (**13**) showed the strongest polarization profile.

**Figure 8:**
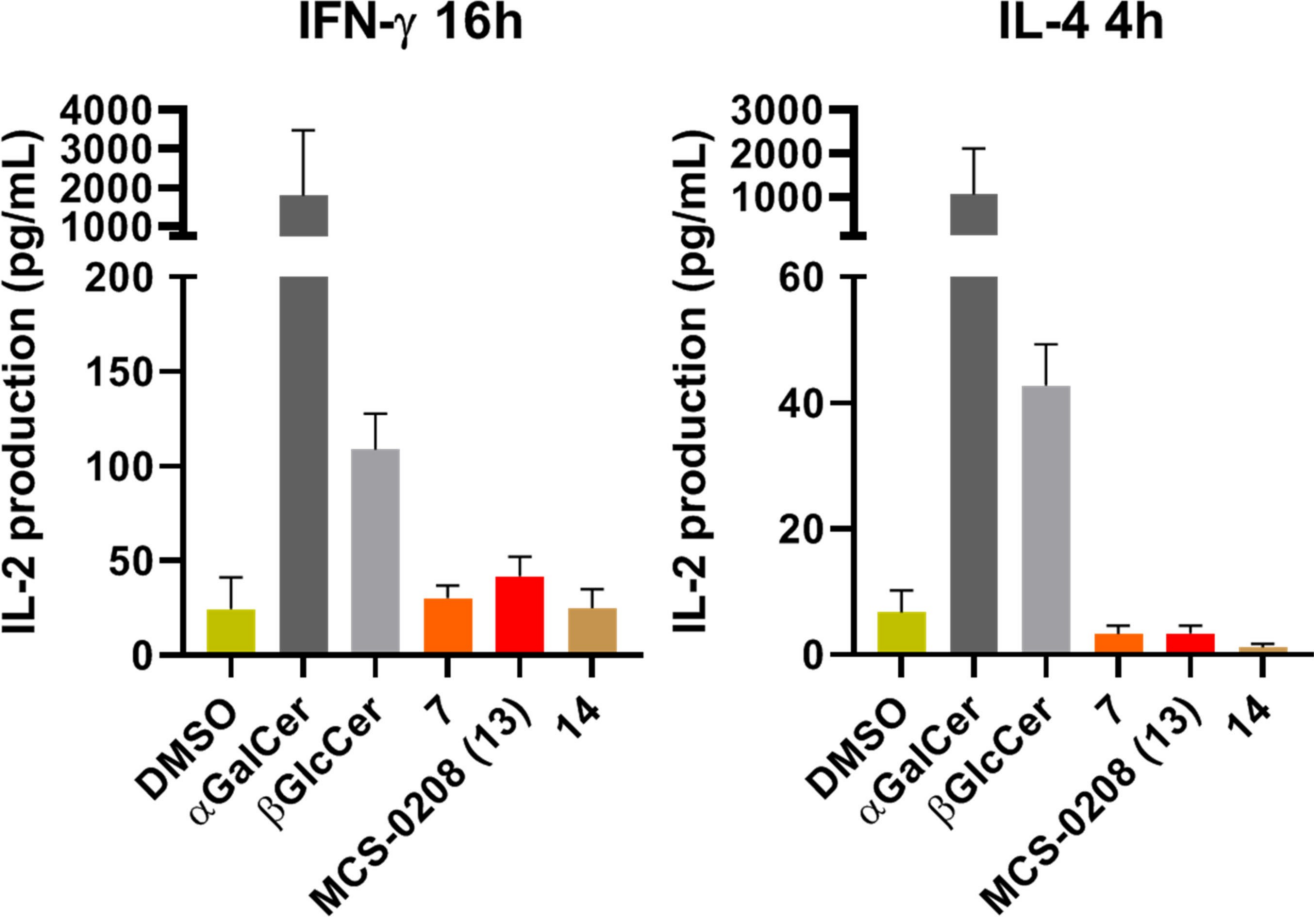
*In vivo* results from BL6 mice injected i. p. Cytokine levels were measured by ELISA assays. Error bars indicatge SD values.

As divergent results in mice and human NKT stimulation have been reported for some compounds, we next tested MCS‐0208 (**13)** in human NKT cell stimulation assays. MCS‐0208 (**13**) induced a TNFα production similar to that of aGalCer (Figure 9 A) suggesting a similar NKT cell stimulation ability and a promising potency. To further explore this hypothesis, IFN‐γ and IL‐4 cytokines levels were measured. At 10ng/mL our compound showed similar IFN‐γ levels than aGalCer however significant lower levels of IL‐4 were detected, pointing to a marked Th1 biased response. These results were unexpected considering the low functionalization of the aromatic ring and therefore the low number of potential contacts of the antigen with the contact surface of CD1d and TCR proteins.

**Figure 9:**
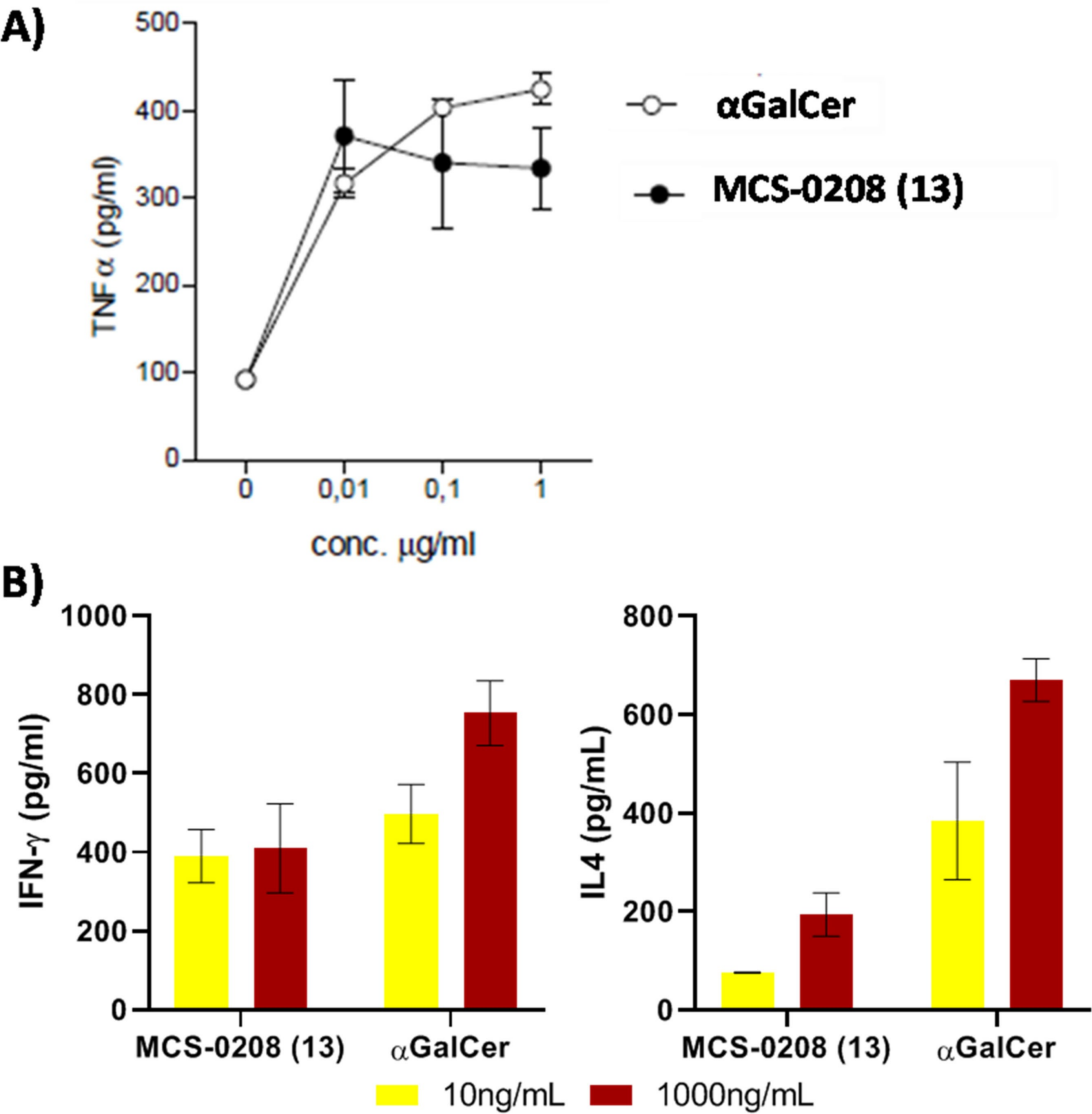
A) *In vitro* TNFα production of human NKT cells. B) *In vitro* IFN‐γ and IL‐4 production of human NKT cells. Purified iNKT from human blood were cocultured with pre‐loaded transfected B‐cells with each antigen in triplicate. Error bars indicate SD values.

To further understand the activity of this unprecedented family, some computational studies were done to explore how this compound could interact with CD1d and the TCR. Initial docking studies were carried out using crystal structure of our potent aminocyciltol HS44 (**3**) and mouse proteins (PDB code: 3RTQ).^[14]^ Protein was prepared following Protein Preparation^[20]^ protocol from Schrödinger,^[21]^ the corresponding grid was centered to the polar head of the aminocyclitol assuming a similar binding mode of the long lipid chains. Due to the high number of rotatable bonds and to speed up the calculation, truncated ligands and positional constraints were used to properly locate each compound into the CD1d cavity and leaving the aromatic moiety unconstrained (or the aminocyclitol moiety of HS44 (**3**) as positive control). This experiment was repeated 10 times to have a good average of potential preferable binding mode. These experiments suggest a binding mode for MCS‐0208 (**13**) with the aromatic ring in parallel to the carbohydrate ring however slightly closer to CD1d than the sugar moiety. Regarding to the only hydroxyl group from MCS‐0208 (**13**), we postulate that it could occupy a similar space to those occupied by the 2’OH and 3’OH of the aminocyclitol (or the sugar mimetic in any case). This binding mode could allow the interaction of this exclusive polar group with two residues essentially involved in the ternary complex formation: Gly96 on the TCR a‐chain and Asp153 on CD1d α‐chain. However, a single H‐Bond would be fromed with Asp153_CD1d_ instead of two that could be observed by the glyco‐mimetic moieties (Figure 10).

**Figure 10:**
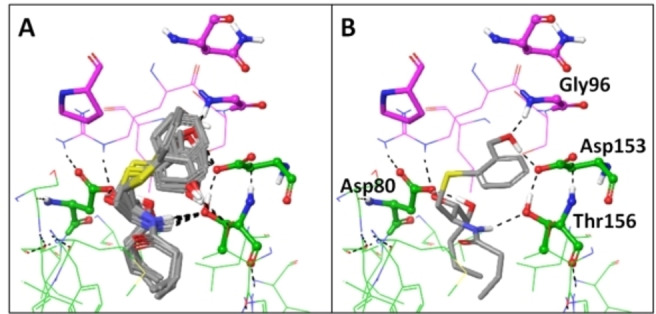
Docking results for MCS‐0208 (**13**) with PDB: 3RTQ crystal structure, A) Superposition of 10 poses obtained after iterative docking jobs B) representative image of the best pose. CD1d carbons are coloured in green while TCR α‐chain carbons are coloured in pink. H‐Bond interactions are coloured in black dashed lines.

To reinforce this hypothesis, a short Molecular Dynamic (MD) simulation was run to explore the system relaxation. The best pose obtained for MCS‐0208 (**13**) was selected as the starting point to run 20 ns MD (300 K, periodic boundary conditions, NPT ensemble) in explicit water, using the Desmond software.^[22]^ Two main parameters were analyzed from MD simulations: ternary complex stability in terms of root‐mean‐square deviation (RMSD) and ligand's H‐Bond formation capacity all along simulation. In this sense, interactions with key residues Asp80_CD1d_, Thr156_CD1d_, Asp153_CD1d_, Asn30_αTCR_, and Gly96_αTCR_ were monitored. Ternary complex structure was mostly stable in all cases with RMSD in the same range of reference HS44 (**3**). H‐Bond analysis showed conserved interactions with Asp80 and Thr156 of CD1d in a similar frequency as HS44 (**3**), only one H‐Bond was observed with Asp153 versus the two observed with HS44 (**3**) (Figure 11). This result was expected as our ligand has a single hydroxyl group. Moreover, interactions with Gly96 on the TCR were lost for MCS‐0208 (**13**) as well as for HS44 (**3**); this result was rather unexpected as this interaction was in the crystal structure analysis of HS44 (**3**)^[14]^ and was also observed on our docking experiments. In a similar manner, in our previous docking experiments MCS‐0208 (**13**) formed this interaction, at least intermittently. A correlation between H‐Bond interaction and the binding affinity together with the potency of NKT cells activation is an attractive hypothesis which is beyond the scope of this preliminary study.

**Figure 11:**
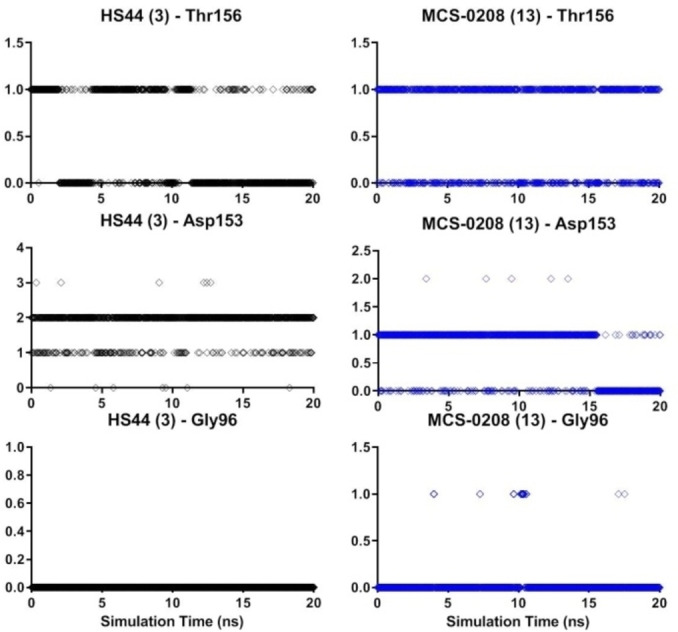
H‐Bond monitoring of HS44 (3) (black dots) and compound 13 (blue dots) during 20 ns MD simulation.

As it could be expected, the number of interactions mediated by “head” group of new thioaryl‐ceramide ligand MCS‐0208 (**13**) decreased due to low level of functionality compared to the aminocyclitol scaffold. To further understand why this compound seems to increase its potency when tested with human cells, preliminary docking studies with human CD1d‐TCR proteins were done (Figure 12). The results point to a new π‐π stacking interaction of the phenyl ring of MCS‐0208 and the unique tryptophan 153 of human CD1d (which is a glycine in mice protein). This interaction could increase the ArCer‐CD1d complex stability and therefore stabilizing the recognition by TCR.

**Figure 12:**
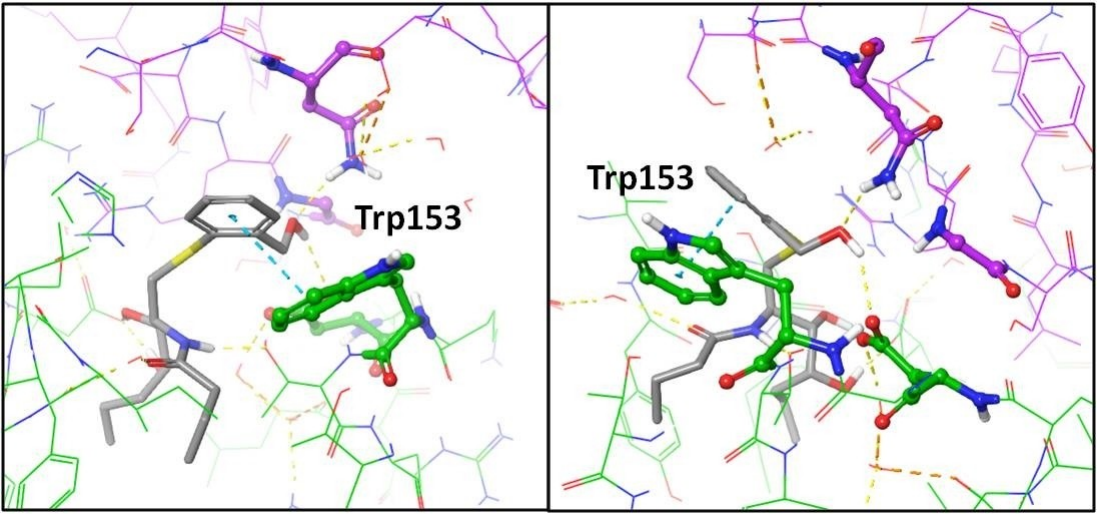
Docking results for MCS‐0208 (13) with PDB: 4NE3^[23]^ crystal structure. Two different views are shown. CD1d carbons are coloured in green while TCR α‐chain carbons are coloured in pink. H‐Bond interactions are coloured in yellow dashed lines and π‐π interactions are coloured in blue.

Overall, these results point to this compound as promising candidate to be further study. More extensive structure‐activity studies could put some light on this new family potential and deeper structural are needed to corroborate the above mentioned structural hypothesis.

## Conclusions

Here we have presented a new NKT cell recognized antigen, MCS‐0208 (**13**), with an unprecedented structure having an *ortho*‐hydroxymethyl substituted phenylthioether as a sugar mimetic in the glycolipid. These results open the possibility of further development for new families of compounds capable to stimulate NKT cells. Considering the low functionalization of this hit compound, we are optimistic of the potential of this new family of NKT cell modulators with a more controllable activity and selectivity. In summary, we have tested a small but representative group of sphingolipid analogues as NKT cell stimulators *in vitro* and the most potent antigens were tested *in vivo*. Surprisingly, the aromatic compound MCS‐0208 (**13**) showed the highest stimulation capacity for both mice and human NKt cells among all tested compounds. Although all of them were weaker antigens than the reference αGalCer, MCS‐0208 (**13**) showed a better Th1 polarizing profile. Taking into account that one of the drawbacks of αGalCer (**1**) is the overstimulation of NKT cells, the immmuno‐stimulatory properties showed by MCS‐0208 (**13**) could be an interesting starting point for the search of new, selective and adjustable molecules as antigens for CD1d‐restrictred NKT cell stimulation with more controlled potency and less disadvantages.

## Conflict of interest

The authors declare no conflict of interest.

## Supporting information

As a service to our authors and readers, this journal provides supporting information supplied by the authors. Such materials are peer reviewed and may be re‐organized for online delivery, but are not copy‐edited or typeset. Technical support issues arising from supporting information (other than missing files) should be addressed to the authors.

Supporting Information
