# Supplementary material for: New Paradigm in NKT Cell Antigens: MCS‐0208 (2‐(Hydroxymethyl)phenylthio‐phytoceramide) – an Aryl‐Phytoceramide Compound with a Single Hydroxyl Group Stimulates NKT Cells
Source: ChemMedChem. 2021 Jun 17;16(16):2491–6. doi: 10.1002/cmdc.202000992 (PMC8453509; doi:10.1002/cmdc.202000992)
Supplement: Supplementary file 1 — Supporting Information [file CMDC-16-2491-s001.pdf]

ChemMedChem

Supporting Information

New Paradigm in NKT Cell Antigens: MCS-0208 (2-(Hydroxymethyl)phenylthio-phytoceramide) – an Aryl-Phytoceramide Compound with a Single Hydroxyl Group Stimulates NKT Cells

Roser Borràs-Tudurí[+], Anna Alcaide[+], Sandrine Aspeslag, Lorena Usero, Carmen Serra, Carme Roura-Mir, Dirk Elewaut, and Amadeu Llebaria*

1. Chemical synthesis:

Materials and Methods:

Nuclear Magnetic Resonance (NMR)

Compound characterization by Nuclear Magnetic Resonance (NMR) spectroscopy was performed with *Variant-Mercury 400 MHz* spectrometer or *Bruker-AVANCEIII-HD 500 MHz* spectrometer equipped with a z-gradient (65.7 G/ cm) inverse TCI cryoprobe. Chemical shifts δ are reported in parts per million (ppm) using residual non-deuterated solvent signal as reference (Chloroform-*d* δ=7.26 ppm (^{1}H), δ=77.16 ppm (^{13}C); DMSO-d_6 δ=2.50 ppm (^{1}H), δ=39.51 ppm (^{13}C), Methanol-d_4 δ=4.87 ppm, δ=3.31 ppm (^{1}H), δ=49.3 ppm (^{13}C)). The following abbreviations were used to designate multiplicities: s=singlet, d=doublet, t=triplet, q=quartet, m=multiplet, q=quintuplet, br=broad, dd=double-doublet, ddd=double-double-doublet. Coupling constant (*J*) was expressed in Hz.

Infrared Spectra (IR)

IR spectra were registered in chloroform solution and recorded with *Thermo Nicolet Avatar 360 FT-IR* Spectrometer.

Melting Point (mp)

Melting points were measured with *Melting Point B-545 (Büchi)*, ramp 0.5 ºC/min with digital temperature measurement.

Specific rotation ($[\alpha]_D$)

$[\alpha]_D$ values were measured in microaperture mode with a *Perkin-Elmer 341* polarimeter with Na/NaI lamp (589 nm) and 1dm of length cell (1 mL of capacity). [α]D are expressed in degrees and calculated as *c* x 100 / (*d* x *m*) where *c* is the concentration of the sample (in g/100 mL), *d* is the optical way (in dm) and *m* is the measured value (mean of 10 measurements).

High-resolution mass spectra (HRMS)

High-resolution mass spectra (HRMS) were analyzed by FIA (flux injected analysis) with Ultrahigh-Performance Liquid Chromatography (UPLC) *Aquity (Waters)* coupled to LCT Premier Orthogonal Accelerated Time of Flight Mass Spectrometer (TOF) (*Waters*). Data from mass spectra were analyzed by electrospray ionization in positive and negative mode. Spectra were scanned between 50 and 1500 Da with values every 0.2 seconds and peaks are given m/z. Data was acquired with MassLynx software version 4.1 (*Waters*). These analyses were performed by the mass spectrometry service of IQAC-CSIC.

Chemical synthesis:

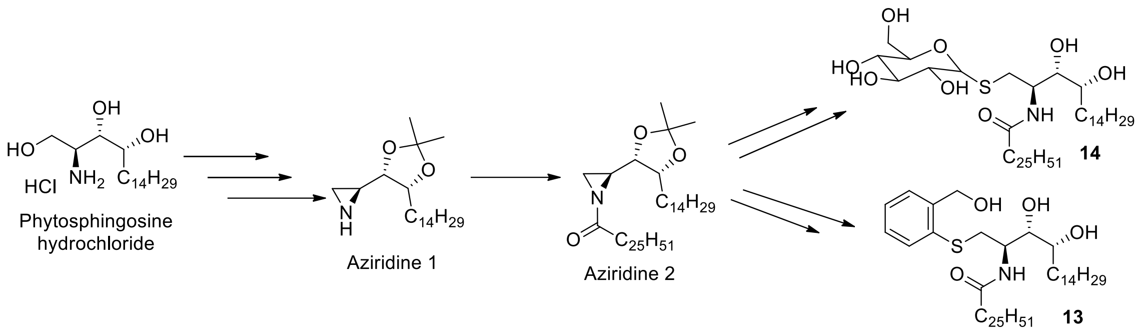

Scheme 1: General synthetic scheme of compounds MCS-0208 (13) and 14

(*S*)-2-((4*S*,5*R*)-2,2-dimethyl-5-tetradecyl-1,3-dioxolan-4-yl)-1-hexacosanoylaziridine (Aziridine 2)

A mixture of cerotic acid (800.0 mg, 2.02 mmol) and thionyl chloride (5 mL, 67.9 mmol) was heated to reflux and stirred for 2 hours. After that, the remaining thionyl chloride was coevaporated with toluene (4 x 5 mL) to afford hexacosanoyl chloride as a pale brown solid (830.0 mg, 99%), which was used in next reaction step without further purification. ^{1}H NMR (400 MHz, $CDCl_3$) δ 2.88 (t, J = 7.3 Hz, 2H), 1.71 (quint, J = 7.3 Hz, 2H), 1.38 – 1.21 (m, 44H), 0.88 (t, J = 6.9 Hz, 3H). IR (film): ν = 2988, 2918, 2851, 1692, 1464, 1367, 1250-1000 cm^{-1}. After, to a solution of (*S*)-2-((4*S*,5*R*)-2,2-dimethyl-5-tetradecyl-1,3-dioxolan-4-yl)aziridine (**Aziridine 1**)[1] (276.0 mg, 0.81 mmol) in dry DCM (20 mL) was added triethylamine (199 μL, 1.43 mmol). The mixture was cooled to -10 ºC and then a solution of hexacosanoyl chloride (4.8 mL, 0.97 mmol) in DCM was added. The reaction mixture was stirred and allowed to warm to room temperature overnight. After, the solvent was removed *in vacuo* to give a crude, which was purified by *flash* chromatography (silica gel, hexane-ethyl acetate 1:0 to 9.5:0.5, gradient) to afford the desired **Aziridine 2** as a white solid (549 mg, 94%). $[\alpha]^{20}_{D}$ -23.2 (*c* 3.6, $CDCl_3$). IR (film): ν = 2988, 2918, 2851, 1692, 1464, 1367, 1248, 1218, 1041 cm^{-1}. ^{1}H NMR (400 MHz, $CDCl_3$) δ 4.20 (dt, J = 8.6, 5.5 Hz, 1H (CH-O)), 3.77 (dd, J = 6.7, 6.2 Hz, 1H (CH-O)), 2.67 – 2.60 (m, 1H (CH-N)), 2.44 (dt, A part of a AB system, J_{AB} = 16.0 Hz, J = 7.6 Hz, 1H (CH_2-CO)), 2.35 (dt, B part of a AB sytem, J_{AB} = 16.0 Hz, J= 7.2 Hz, 1H (CH_2-CO)) 2.27 (dd, A part of a AB system, J = 5.9 Hz, J_{AB} = 0.9 Hz, 1H (CH_2-N)), 2.22 (dd, J = 3.2 Hz, J_{AB} = 0.9 Hz, 1H (CH_2-N)), 1.86 – 1.70 (m, 2H), 1.67 – 1.57 (m, 2H), 1.55 – 1.43 (m, 3H), 1.46 (s, 3H (CH_3)) 1.41 – 1.19 (m, 65H), 1.34 (s, 3H (CH_3)), 0.88 (t, J = 6.8 Hz, 6H (2CH_3)). ^{13}C NMR (101 MHz, $CDCl_3$) δ 185.8 (C=O), 108.2 (C), 78.6 (CH-O), 78.0 (CH-O), 36.9 (CH_2-CO), 35.2 (CH-N), 32.1 (CH_2), 29.9 (CH_2), 29.8 (3CH_2), 29.7 (CH_2), 29.5 (2CH_2), 28.5 (CH_2-N), 28.1 (CH_3), 26.7 (CH_2), 25.5 (CH_3), 25.1 (CH_2), 22.9 (CH_2), 14.3 (2CH_3). HRMS calculated for $C_{47}H_{92}NO_3$: 718.7077 $[M+H]^+$; found: 718.7103. mp: 62-63 ºC.

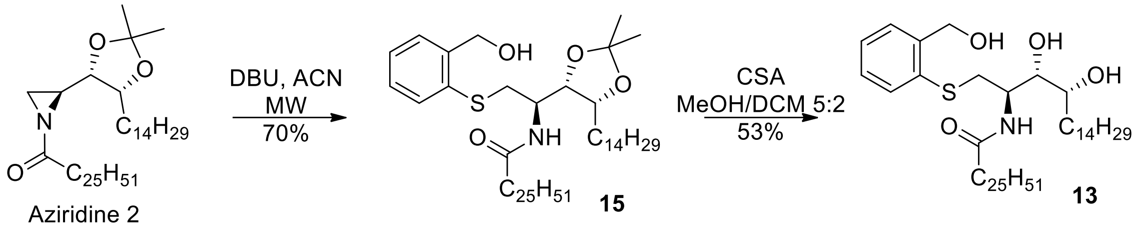

Scheme 2: Shynthesis of compound MCS-0208 (**13**)

General procedure 1 – Microwave-enhanced nucleophilic ring-opening reaction of acylated aziridine derivatives with thiols

In a 10 mL vessel, a solution of aziridine derivative and thiol in MeCN was prepared. After, DBU was added and the mixture was stirred for 3 minutes under nitrogen. The vessel was sealed with a septum and placed into the microwave cavity. The microwave source was then turned on. Constant microwave irradiation with simultaneous air-cooling were used during the entire reaction. The evolution of the reaction was monitored by TLC. When judged complete, the reaction mixture was cooled to room temperature and the solvent was removed under reduced pressure to yield the crude product. *Flash* chromatography on silica gel using a mixture of hexane and ethyl acetate afforded the pure ring-opened products.

***N*-((2*R*,3*S*,4*R*)-1-((2-(hydroxymethyl)phenyl)thio)-3,4-isopropylidenedioxy-octadecan-2-yl) hexacosanamide (15)**

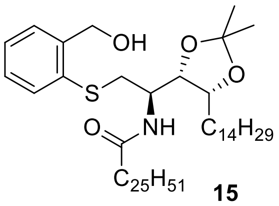

15

Product **15** was synthesized from **Aziridine 2** (38.7 mg, 0.054 mmol), 2-mercaptobenzyl alcohol (9.8 mg, 0.070 mmol) and DBU (9 μL, 0.060 mmol) in MeCN (500 μL), according to *general procedure 1* (150 W, 689.5 kPa, 100ºC) to give complete conversion after 135 min. After purification by *flash* chromatography (silica gel, hexane-ethyl acetate 9.5:0.5 to 8:2, gradient), pure alcohol **15** was isolated as a white solid (32.5 mg, 70%). $[\alpha]^{20}_{D}$ -8.9 (*c* 1.04, $CDCl_3$). IR (film): ν = 3276, 2953, 2917, 2849, 1646, 1544, 1469 cm^{-1}. ^1H NMR (400 MHz, $CDCl_3$) δ 7.55 – 7.50 (m, $1H_{ar}$), 7.45 – 7.40 (m, $1H_{ar}$), 7.29 – 7.21 (m, $2H_{ar}$), 5.88 (d, *J* = 9.2 Hz, 1H (NH)), 4.97 (d, A part of a AB sytem, J_{AB} = 12.3 Hz, 1H (CH_2-O)), 4.65 (d, B part of a AB system, J_{AB} = 12.3 Hz, 1H (CH_2-O)), 4.26 – 4.18 (m, 1H (CH-N)), 4.15 (t, *J* = 6.2 Hz, 1H (CH-O (3))), 4.10 – 4.03 (m, 1H (CH-O (4))), 3.30 (dd, A part of a AB system, J_{AB} = 14.2, 2.8 Hz, 1H (CH_2-S)), 3.09 (dd, B part of a AB system, J_{AB} = 14.2, 7.0 Hz, 1H (CH_2-S)), 1.97 – 1.87 (m, 2H), 1.55 – 1.35 (m, 3H), 1.37 (s, 3H (CH_3)), 1.35 – 1.14 (m, 69H), 1.33 (s, 3H (CH_3)), 0.88 (t, *J* = 6.9 Hz, 6H ($2CH_3$)). ^{13}C NMR (101 MHz, $CDCl_3$) δ 173.2 (C=O), 142.2 (C_{ar}), 134.8 (C_{ar}), 133.0 (CH_{ar}), 129.8 (CH_{ar}), 128.8 (CH_{ar}), 127.8 (CH_{ar}), 108.3 (C), 78.2 (CH-O), 77.8 (CH-O), 63.6 (CH_2-O), 49.2 (CH-N), 38.4 (CH_2-S), 36.7 (CH_2-CO), 32.1 (CH_2), 29.9 ($2CH_2$), 29.8 ($3CH_2$), 29.7 (CH_2), 29.6 (CH_2), 29.5 ($3CH_2$), 29.0 (CH_2), 27.6 (CH_3), 26.8 (CH_2), 25.6

(CH_3), 25.6 (CH_2), 22.9 (CH_2), 14.3 ($2CH_3$). HRMS calculated for $C_{54}H_{100}NO_4S$: 858.7373 $[M+H]^+$; found: 858.7336. mp: 84-86 ºC

***N*-((2*R*,3*S*,4*R*)-3,4-dihydroxy-1-((2-(hydroxymethyl)phenyl)thio)octadecan-2-yl)hexacosanamide (MCS-0208, 13)**

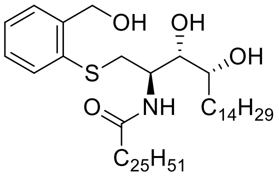

MCS-0208 (13)

To a solution of product **15** (24.5 mg, 0.029 mmol) in a mixture (2 mL) of MeOH-DCM (5:2), was added CSA (13.3 mg, 0.057 mmol). The resulting suspension was heated to 30 ºC and stirred overnight. Then, the solvent was removed under reduced pressure to give a crude which was redissolved in AcOEt (20 mL) and it was washed with 1N aq. NaOH (3 x 20 mL). After, the organic layer was dried over $MgSO_4$, filtered and the solvent was removed under reduced pressure to give a white solid, which was purified by *flash* chromatography (silica gel, hexane-ethyl acetate 8:2) to afford pure triol **13** as a white solid (12.4 mg, 53%). $[\alpha]^{20}_D$ -12.7 (*c* 0.55, $CHCl_3$). IR (film): ν = 3303, 2953, 2918, 2850, 1637, 1468, 1068 cm^{-1}. ^1H NMR (400 MHz, 1:1 $CDCl_3$:CD_3OD, chemical shifts are referred to the singlet at δ = 7.26 ppm of $CDCl_3$) δ 7.50 – 7.44 (m, 1H_{ar}), 7.41 – 7.34 (m, 1 H_{ar}), 7.25 – 7.17 (m, 2 H_{ar}), 6.88 (d, *J* = 8.6 Hz, 1H (NH)), 4.84 (d, A part of a AB system, J_{AB} = 12.1 Hz, 1H (CH_2-O)), 4.65 (d, B part of a AB system, J_{AB} = 12.1 Hz, 1H (CH_2-O)), 4.10 – 3.97 (m, 1H (CH-N)), 3.58 (dd, *J* = 5.6, 5.2 Hz, 1H (CH-O (3))), 3.49 – 3.41 (m, 1H (CH-O (4))), 3.30 (dd, A part of a AB system, J_{AB} = 14.0 Hz, *J* = 3.4 Hz, 1H (CH_2-S)), 3.15 (dd, B part of a AB system, J_{AB} = 14.0 Hz, *J* = 8.4 Hz, 1H (CH_2-S)), 2.38 (br s, 3H (OH)), 1.95 (t, *J* = 7.8 Hz, 2H (CH_2-CO)), 1.53 – 1.40 (m, 3H), 1.33 – 1.14 (m, 69H), 0.84 (t, *J* = 6.8 Hz, 6H ($2CH_3$)). ^{13}C NMR (101 MHz, 1:1 $CDCl_3$:CD_3OD, chemical shifts are referred to the centre line of the triplet at δ = 77.0 ppm of $CDCl_3$) δ 174.8 (C=O), 141.8 (C_{ar}), 134.8 (C_{ar}), 132.7 (CH_{ar}), 129.7 (CH_{ar}), 128.7 (CH_{ar}), 127.5 (CH_{ar}), 75.6 (CH-O (3)), 72.6 (CH-O (4)), 63.2 (CH_2-O), 51.7 (CH-N), 36.4 (CH_2-CO), 35.8 (CH_2-S), 32.7 (CH_2), 32.0 (CH_2), 29.8, (CH_2) 29.7, (CH_2) 29.6 (CH_2), 29.5 (CH_2), 29.4 ($2CH_2$), 25.9 (CH_2), 25.7 (CH_2), 22.8 (CH_2), 14.2 ($2CH_3$). HRMS calculated for $C_{51}H_{96}NO_4S$: 818.7060 $[M+H]^+$; found: 818.7062. mp: 106-108 ºC

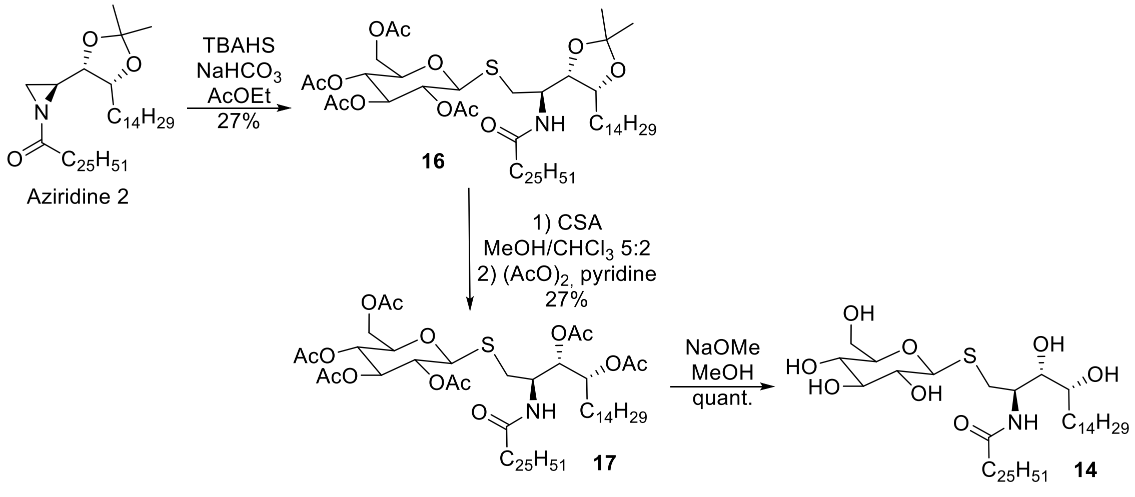

Scheme 3: Shynthesis of compound 14

(2*R*,3*S*,4*R*)-tetra-*O*-acetyl-1-β-D-glucopyranosylthio-2-hexacosanamido-3,4-isopropylidene dioxyoctadecane (16)

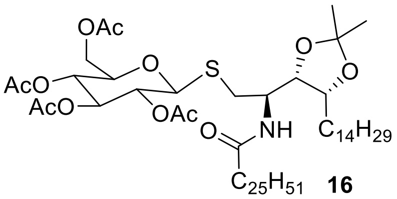

16

To a solution of **aziridine 2** (41.0 mg, 0.057 mmol) and 1-Thio-β-D-glucose tetraacetate (49.9 mg, 0.14 mmol) in a previously degassed mixture of AcOEt (440 μL) and 1M aq. $NaHCO_3$ (440 μL), was added TBAHS (78.0 mg, 0.23 mmol). The resulting mixture was irradiated in a microwave reactor (150 W, 689.5 kPa, 40 ºC) for 2 hours. After that, the reaction mixture was diluted with AcOEt (20 mL) and it was washed with sat. aq. $NaHCO_3$ (3 x 20 mL). The organic layer was dried over $MgSO_4$, filtered and the solvent was removed under reduced pressure to give a crude which was purified by *flash* chromatography (silica gel, hexane-ethyl acetate 9.5:0.5 to 7:3, gradient) to afford pure product **16** as a white waxy solid (16.7 mg, 27%). ^{1}H NMR (400 MHz, $CDCl_3$) δ 5.75 (d, *J* = 8.8 Hz, 1H (NH)), 5.22 (t, *J* = 9.4 Hz, 1H (CH-O (3'))), 5.06 (t, *J* = 9.8 Hz, 1H (CH-O (4'))), 4.99 (t, *J* = 9.6 Hz, 1H (CH-O (2'))), 4.52 (d, *J* = 10.1 Hz, 1H (S-CH-O)), 4.25 (dd, A part of a AB system, J_{AB} = 12.5 Hz, *J*= 4.9 Hz, 1H (CH_2-O)), 4.22 – 4.17 (m, 1H (CH-N)), 4.17 – 4.09 (m, 3H (B part of a CH_2-O AB system, CH-O (3) and CH-O (4))), 3.69 (ddd, *J* = 10.1, 4.8, 2.3 Hz, 1H (CH-O (5'))), 3.02 (dd, A part of a AB system, J_{AB} = 13.9 Hz, *J* = 3.4 Hz, 1H (CH_2-S)), 2.86 (dd, B part of a AB system, J_{AB} = 13.9 Hz, *J* = 7.1 Hz, 1H (CH_2-S)), 2.15 (td, *J* = 7.1, 3.1 Hz, 2H (CH_2-CO)), 2.09 (s, 3H (CH_3)), 2.06 (s, 3H (CH_3)), 2.03 (s, 3H (CH_3)), 2.01 (s, 3H (CH_3)), 1.65 – 1.56 (m, 4H), 1.56 – 1.49 (m, 3H), 1.45 (s, 3H (CH_3)), 1.38 – 1.16 (m, 66H), 1.33 (s, 3H (CH_3)), 0.88 (t, *J* = 6.8 Hz, 6H (2CH_3)). ^{13}C NMR (101 MHz, $CDCl_3$) δ 172.7 (NH-C=O), 170.8 (C=O), 170.2 (C=O), 169.7 (C=O), 169.5 (C=O), 108.1 (C), 83.9 (S-CH-O), 78.3 (CH-O), 77.9 (CH-O), 76.3 (CH-O (5')), 73.9 (CH-O (3')), 70.0 (CH-O (2')), 68.4 (CH-O (4')), 62.1 (CH_2-O), 47.8 (CH-N), 36.9 (CH_2-CO), 32.8 (CH_2-S), 32.1 (CH_2), 29.9 (CH_2), 29.8 (4CH_2), 29.7 (2CH_2), 29.6 (2CH_2), 29.5 (2CH_2), 29.2 (CH_2), 27.7 (CH_3), 27.0 (CH_2), 25.8 (CH_2), 25.6 (CH_3), 22.8 (CH_2), 20.9 (2CH_3), 20.7 (2CH_3), 14.3 (2CH_3).

(2*R*,3*S*,4*R*)-tetra-*O*-acetyl-1-β-D-glucopyranosylthio-3,4-diacetoxy-2-hexacosanamido octadecane (17)

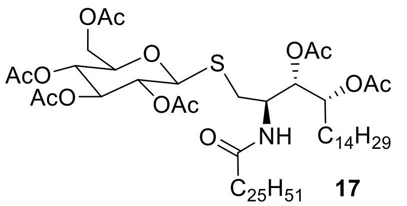

To a solution of **16** (16.7 mg, 0.015 mmol) in a mixture (2 mL) of dry MeOH-$CHCl_3$ (5:2), was added CSA (7.2 mg, 0.031 mmol). The resulting suspension was heated to 30 ºC and stirred overnight. After, the solvent was removed under reduced pressure to give a crude which was redissolved in pyridine (560 μL) and acetic anhydride (22 μL, 0.23 mmol) was added. The mixture was stirred at room temperature overnight. Then, the reaction mixture was cooled to 0 ºC and MeOH was added dropwise. The solvent was removed under reduced pressure to give a crude that was redissolved in AcOEt and washed with water (2 x 20 mL), sat. aq. $NaHCO_3$ (2 x 20 mL) and 1N aq. HCl (2 x 20mL). The organic layer was dried over $MgSO_4$, filtered and the solvent was removed under reduced pressure to give a crude, which was purified by *flash* chromatography (silica gel, hexane-ethyl acetate 9.5:0.5 to 7:3, gradient) to get product **17** as a colorless oil (4.6 mg, 27%). ^{1}H NMR (400 MHz, $CDCl_3$) δ 6.00 (d, *J* = 9.0 Hz, 1H), 5.22 (t, *J* = 9.4 Hz, 1H), 5.11 – 5.02 (m, 2H), 5.02 – 4.92 (m, 2H), 4.43 (d, *J* = 10.1 Hz, 1H), 4.41 – 4.35 (m, 1H), 4.25 (dd, *J* = 12.5, 4.6 Hz, 1H), 4.18 (dd, *J* = 12.5, 2.3 Hz, 1H), 3.75 (ddd, *J* = 10.1, 4.5, 2.3 Hz, 1H), 3.02 (dd, *J* = 13.8, 4.1 Hz, 1H), 2.69 (dd, *J* = 13.7, 7.3 Hz, 1H), 2.19 (t, *J* = 7.6 Hz, 2H), 2.11 (s, 3H), 2.09 (s, 3H), 2.06 (s, 3H), 2.05 (s, 3H), 2.03 (s, 3H), 2.01 (s, 3H), 1.71 – 1.54 (m, 8H), 1.35 – 1.21 (m, 64H), 0.88 (t, *J* = 6.8 Hz, 6H).

(2*R*,3*S*,4*R*)-1-β-D-glucopyranosylthio-2-hexacosanamido-3,4- dihydroxyoctadecane (14)

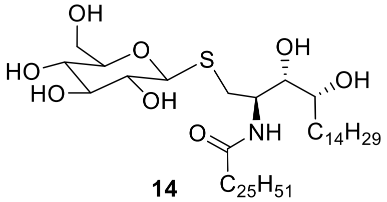

To a solution of adduct **17** (4.6 mg, 0.004 mmol) in MeOH (500 μL), was added a 25% methanolic solution of sodium methoxide (13 μL, 0.057mmol) and the resulting mixture was stirred at room temperature overnight. Then, the solvent of the reaction mixture was removed *in vacuo* to give a crude compound that was used without further purification in biological studies. ^{1}H NMR (500 MHz, Pyridine-*d*$_5$) δ 8.69 (d, *J* = 8.8 Hz, 1H), 7.39 (d, *J* = 5.0 Hz, 1H), 7.32 (d, *J* = 3.3 Hz, 1H), 7.14 (s, 1H), 6.48 (t, *J* = 6.1 Hz, 1H), 6.71 (d, *J* = 5.8 Hz, 1H), 6.07 (d, *J* = 6.9 Hz, 1H), 5.35 – 5.27 (m, 1H), 5.20 (d, *J* = 9.1 Hz, 1H), 4.57 (ddd, *J* = 11.8, 6.0, 2.3 Hz, 1H), 4.40 (q, *J* = 6.0 Hz, 1H), 4.32 (dt, *J* = 11.9, 6.1 Hz, 1H), 4.25 – 4.14 (m, 4H), 4.00 (d, *J* = 4.2 Hz, 1H), 3.97 (d, *J* = 4.7 Hz, 1H), 3.98 – 3.96

(m, 1H), 3.51 (dd, *J* = 13.9, 9.3 Hz, 1H), 2.58 – 2.46 (m, 2H), 1.96 – 1.83 (m, 2H), 1.30 (dd, *J* = 28.3, 7.2 Hz, 66H), 0.88 (dt, *J* = 7.1, 3.5 Hz, 6H); ^{13}C NMR (126 MHz, Pyr) δ 173.78, 87.06, 82.92, 80.26, 77.33, 74.90,72.17, 63.11, 63.07, 52.18, 37.06, 34.38, 31.15, 31.14, 30.03, 26.63, 26.57, 22.98, 14.38; HRMS calculated for $C_{50}H_{99}NO_8SNa$: 896.6989 $[M+Na]^+$; found: 896.6818.

2. Biological studies of the analogs as antigens for CD1d-restricted iNKT cells

In vitro **and** ***in vivo*** **experiments with mice:**

Synthetic and commercial sphingolipid analogs

Sphingolipid analogs were synthesized at the MCS group, which belongs to the IQAC at the Spanish National for Research Council (CSIC). α-GalCer was kindly provided M.Tsuji (Aaron Diamond AIDS Research Center, NY, USA) and the NIH Tetramer Core Facility. β-GlucCer was purchased from Avanti Polar Lipids. Sphingolipid analogs were dissolved in pure DMSO (Sigma) at 1 mg/mL concentration and stored at -20 ºC. Analogs were solubilized by adding PBS (Invitrogen) and vehicle (96mg/mL sucrose, 10mg/mL sodium deoxycholate, 0.05% Tween 20), warming to 80 ºC for 20 minutes and sonicating for 10 minutes at the same temperature.

Cell Lines

The murine iNKT hybridoma 2C12 (Vα14Vβ88.2b) was provided by L. Brossay (Brown University, Providence, RI, USA). Cells were cultured in DMEM (Sigma) supplemented with 10% fetal calf serum (Invitrogen), 1% glutamine (Sigma), 1% penicillin streptomycin (Sigma) and β-mercaptoethanol (Sigma) (called cDMEM hereafter).

Isolation and expansion of BMDCs

BMDCs were isolated from the mouse bone marrow and grown with GM-CSF during 7 days. Cells were cultured in RPMI (Gibco) supplemented with 10% fetal calf serum (Invitrogen), 1% glutamine (Sigma), 1% penicillin streptomycin (Sigma) and β-mercaptoethanol (Sigma).

Mice

C57BL/6 mice were in house bred (in accordance with the general recommendations for animal breeding and housing) or purchased from the Harlan Laboratory, Jα18-knockout mice on the C57BL/6 background were kindly provided by M. Taniguchi (RIKEN, Tsurumi, Yokohama, Japan). All animal experiments were approved by the local Ethical Committee of Laboratory Animals Welfare of Ghent University (reference ECD15/75). Mice used for experiments were between 6 and 9 weeks old.

In vitro and i*n vivo* activation of iNKT cells

For *in vitro* stimulation a coculture between murine iNKT hybridoma cells (grown at $5.10E^4$ cells/well in 96-well plates) and glycolipid pulsed BMDCs ($10E^5$ cells/well) was set up. BMDCs were stimulated with the glycolipid at 100ng/mL, 500 ng/mL or 1000 ng/mL during 20h. Coculture lasted for 16 hours and hereafter supernatant was discarded. Levels of murine IL-2 in the supernatant were determined by ELISA.

For *in vivo* activation of iNKT cells C57BL/6 mice were intraperitoneally injected with 5µg glycolipid (dissolved in 500µL PBS). They were bled at 4h and 16h after injection and serum was analyzed for IFN-γ and IL-4 with ELISA.

***In vitro* assays with human cells:**

Cells used as APC and NKT

C1R-CD1d$^+$ were used as APC. C1R-CD1d$^+$ consist on a transfected C1R cells with CD1d proteins while lacks surface HLA A and B antigens typical from human B-cell linage.

Human NKT cells were purified from healthy donor peripheral blood anonymized samples obtained from the Blood and Tissue Bank of Barcelona with the approval of the Scientific Committee and Scientific Director of the Blood Bank Biobank (July 15th 2016). The study was approved by the Ethics Committee on Animal and Human Experimentation of the Universitat Autònoma de Barcelona (CEEAH project# 3112).

iNKT cells were isolated from peripheral blood lymphocytes samples using density gradient using Ficoll (Lymphoprep). iNKT cells were specifically isolated from the rest of lymphocytes using an Automacs system based on human Anti-iNKT Microbeads kit (Miltenyi Biotec). This kit is based on monoclonal antibodies anti- Vα24Jα18 able to generate two cell populations: those ones with Vα24Jα18$^+$ phenotype (iNKT-cells) and those ones without it (Vα24Jα18$^-$) corresponding to another cell population.

In vitro stimulation assay

For in vitro stimulation, three cell types were used: C1R-CD1d$^+$ cells as APC, PBMs as feeders and iNKT cells as "*reactive*" cells. C1R-CD1d$^+$ cells were incubated at 90000 cells/eppendorff and each analogue at different concentrations during 1 hour at 37ºC and then they were irradiated at 45 Gy. Equally, PBMs were also irradiated at 30 Gy. After that, C1R-CD1d$^+$ cells loading different analogues were plated at 30000 cells/well in 96-well plate together with PBMs at 40000 cells/well and co-cultured with purified iNKT at 10000 cells/well. αCD3/CD28 conjugated beads (Dynabeeds, Lite Technologies) were used as positive control of the assay. Assay medium used was IMDM (Iscove's Modified Dolbecco's Medium) complemented with 8% of human serum, L-Glutamine (2 mM), Penicillin (100 U/mL) and Streptomycin (100 μg/mL). αGalCer (**2**) was used as positive control at 100 ng/mL. Aromatic-ceramide compounds were resuspended in 100% of DMSO at concentration of 1 mg/mL. Less than 5% of DMSO in cellular assay was present in our cultures, a concentration described not toxic for cells. Levels of human IL-4, TNF-α and IFN-γ in the supernatants were determined by ELISA.

3. Computational studies of new aromatic-ceramide derivatives

All the computational work was carried out with the Schrödinger Suite 201420, through its graphical interface Maestro[2]. Coordinates of TCR-Ag-CD1d (PDB code 3RTQ[3] and 4EN3[4]) were obtained from the Protein Data Bank[5] web site. Protein X-ray structures were prepared using

the Protein Preparation Wizard[6] included in Maestro to remove solvent molecules and ions, adding hydrogens, setting protonation states and minimizing energies. Ligands were set up with the LigPrep module[7] included in Maestro to generate ionization states, ring conformations and geometry optimization previous to use. The program MacroModel[8], its default force field OPLS 2005 (a modified version of the OPLS-AA force field)[9] and GB/ SA water solvation conditions were used for all energy calculations. The program Glide[10] was used for the docking calculations using the default XP precision settings except for a setting of 100 000 poses per ligand for the initial phase of docking, a scoring window of 200 for keeping initial poses, and a limit of 800 poses per ligand for energy minimization. Selected ligands were submitted under Molecular Dynamics simulations using Desmond software included in Schrödinger Suite at 20ns, considering explicit water at 300K under Periodic Boundary conditions (NPT ensemble) using OPLS 2005 force field[9]. Simulation systems for molecular dynamics were built using the System Builder of the Maestro-Desmond interface[11], which automatically assigns parameters to all atom. Each CD1d-Ag-TCR complex was immersed in an orthorhombic box (sides at 10 Å of the closest solute atom) of TIP3P water with enough Cl- anions to achieve neutrality (~35000 water molecules, ~106000 atoms in total). Systems were relaxed by minimization (initial steepest descent followed by LBFGS minimization), first with the solute restrained and then without restrains, until a gradient threshold of 0.1 kcal $mol^{-1}Å^{-1}$ was reached. Then, they were heated stepwise up to 300 K with short MD runs under periodic boundary conditions (PBC) (25 ps at 0.1, 10, 100 and 300 K), and equilibrated for 2 ns at the same temperature and 1.0 bar, in the NPT ensemble. Production MD simulations (20 ns, 2 fs timestep) were performed under the same conditions (PBC, NPT ensemble, 300 K and 1.0 bar) using the Nose-Hoover thermostat method[12,13] with a relaxation time of 1.0 ps and the Martyna-Tobias-Klein barostat method[14] with isotropic coupling and a relaxation time of 2 ps. Integration was carried out with the RESPA integrator[13] using time steps of 2.0, 2.0, and 6.0 fs for the bonded, short range and long range interactions, respectively. A cut-off of 9.0 Å was applied to van der Waals and short-range electrostatic interactions, while long-range electrostatic interactions were computed using the smooth particle mesh Ewald method with an Ewald tolerance of 10^{-9}[15,16]. Bond lengths to hydrogen atoms were constrained using the Shake algorithm[17]. Coordinates were saved every 20 ps, hence 1000 snapshots were obtained from each MD run. The Simulation Event Analysis application included in the Desmond-Maestro interface was used to analyze the trajectories.

4. NMR spectrum of new intermediates and compounds.

^{1}H-NMR spectrum of *N*-((2*R*,3*S*,4*R*)-1-((2-(hydroxymethyl)phenyl)thio)-3,4-isopropylidenedioxy-octadecan-2-yl)hexacosanamide (**15**, 400 MHz, CDCl3)

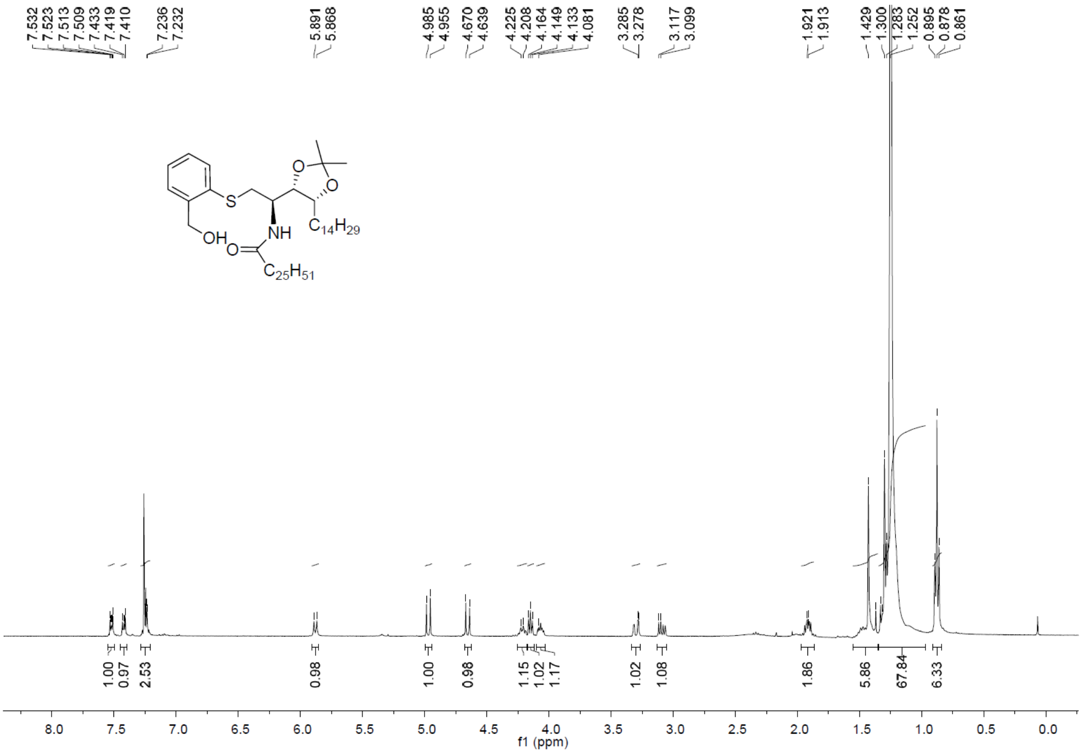

^{13}C-NMR spectrum of *N*-((2*R*,3*S*,4*R*)-1-((2-(hydroxymethyl)phenyl)thio)-3,4-isopropylidenedioxy-octadecan-2-yl)hexacosanamide (**15**, 101 MHz, CDCl3)

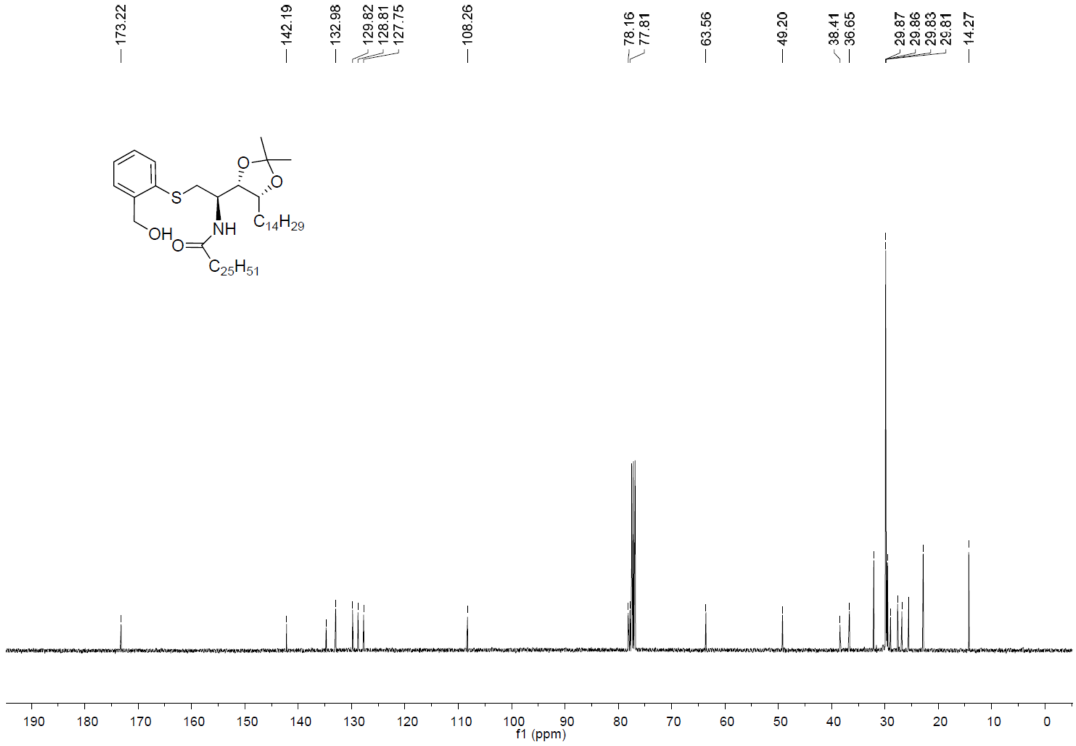

^{1}H-NMR spectrum of *N*-((2*R*,3*S*,4*R*)-3,4-dihydroxy-1-((2-(hydroxymethyl)phenyl)thio) octadecan-2-yl)heptacosanamide (**MCS-0208 - 13**, 400 MHz, 1:1 $CDCl_3$:CD_3OD)

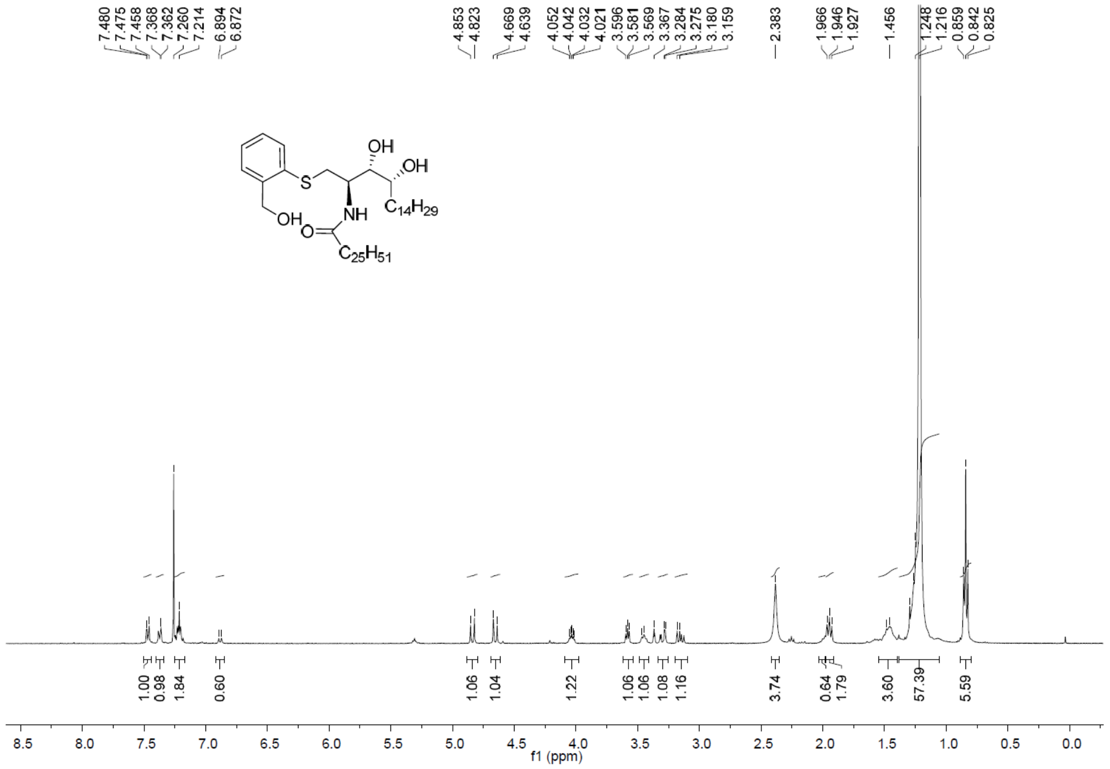

^{13}C-NMR spectrum of *N*-((2*R*,3*S*,4*R*)-3,4-dihydroxy-1-((2-(hydroxymethyl)phenyl)thio) octadecan-2-yl)heptacosanamide (**MCS-0208 - 13**, 101 MHz, 1:1 $CDCl_3$:CD_3OD)

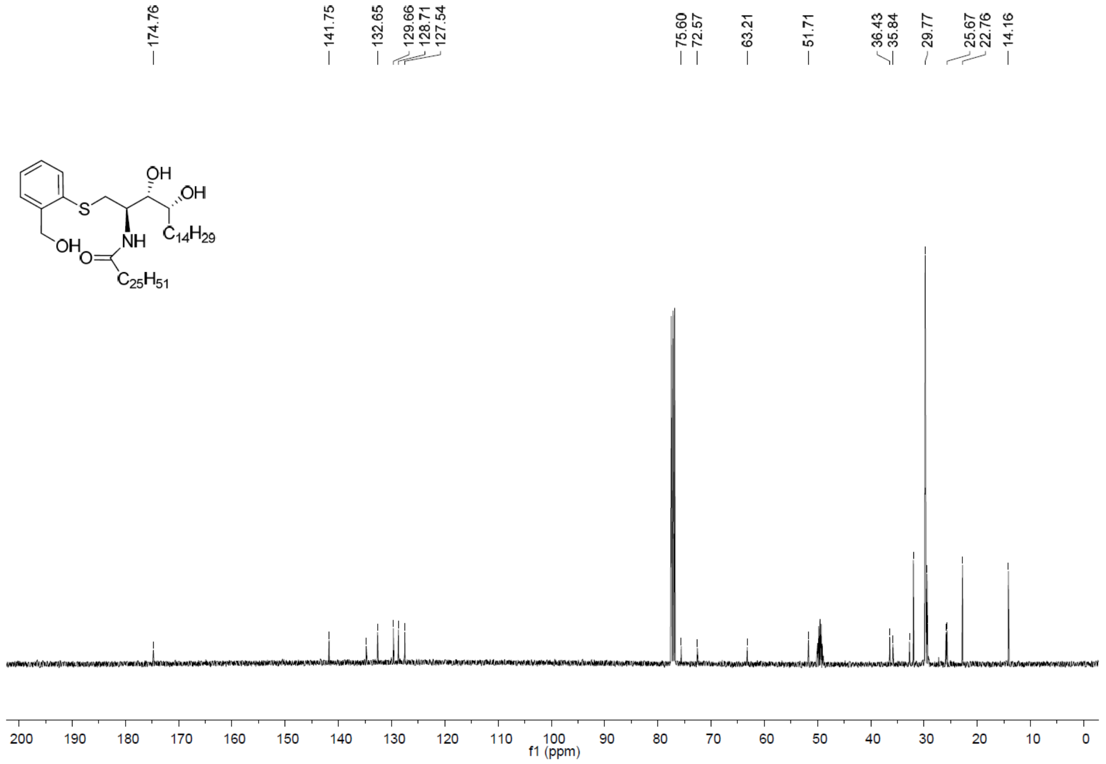

^{1}H-NMR spectrum of (2*R*,3*S*,4*R*)-tetra-*O*-acetyl-1-β-D-glucopyranosylthio-2-hexacosanamido-3,4-isopropylidene dioxyoctadecane (**16**, 400 MHz, $CDCl_3$)

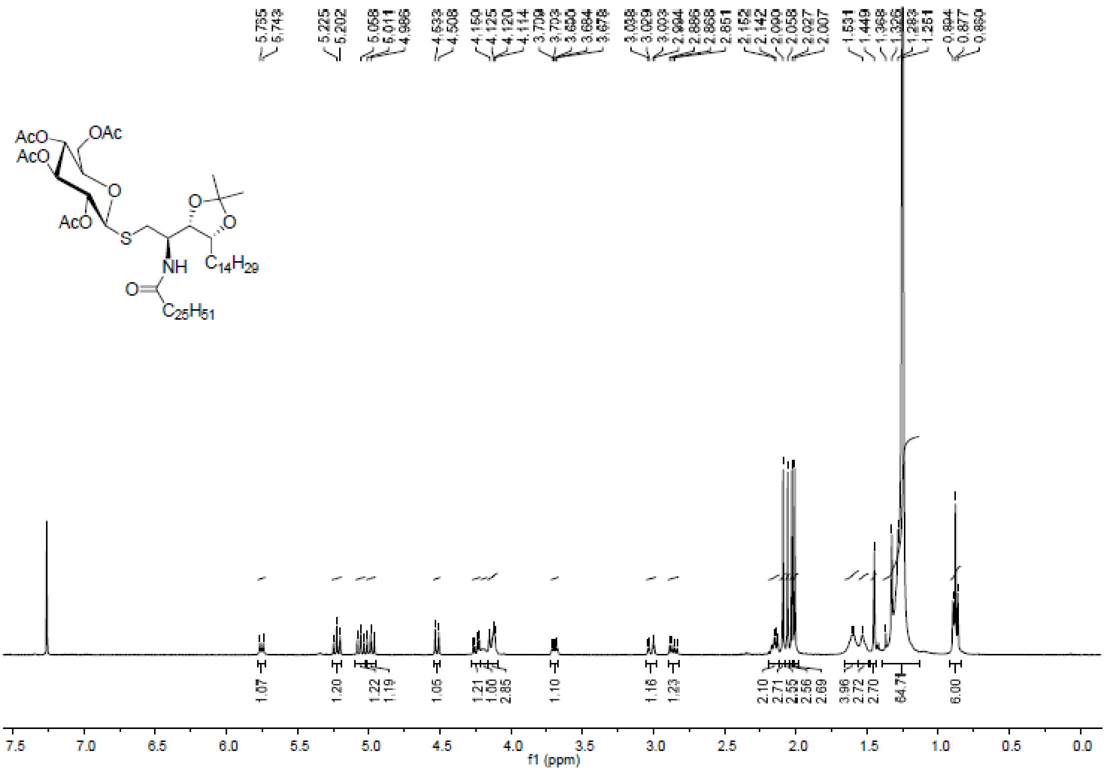

^{13}C-NMR spectrum of (2*R*,3*S*,4*R*)-tetra-*O*-acetyl-1-β-D-glucopyranosylthio-2-hexacosanamido-3,4-isopropylidene dioxyoctadecane (**16**, 101 MHz, $CDCl_3$)

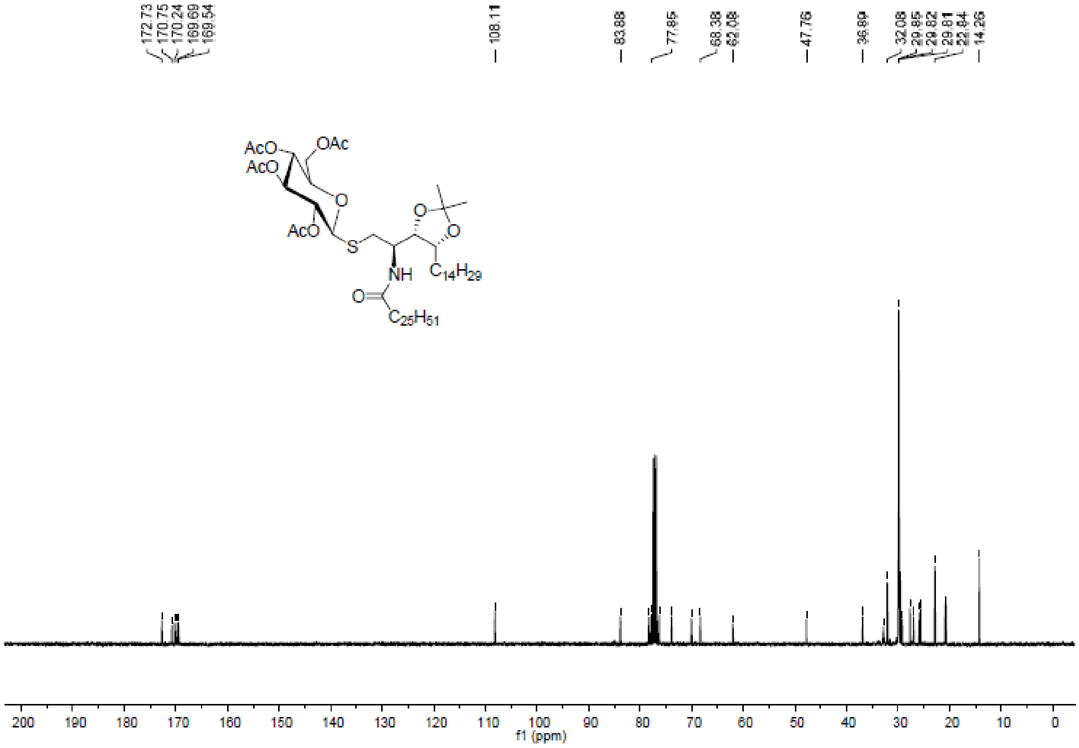

^{1}H-NMR spectrum of (2*R*,3*S*,4*R*)-tetra-*O*-acetyl-1-β-D-glucopyranosylthio-3,4-diacetoxy-2-hexacosanamidooctadecane (**17**, 400 MHz, $CDCl_3$)

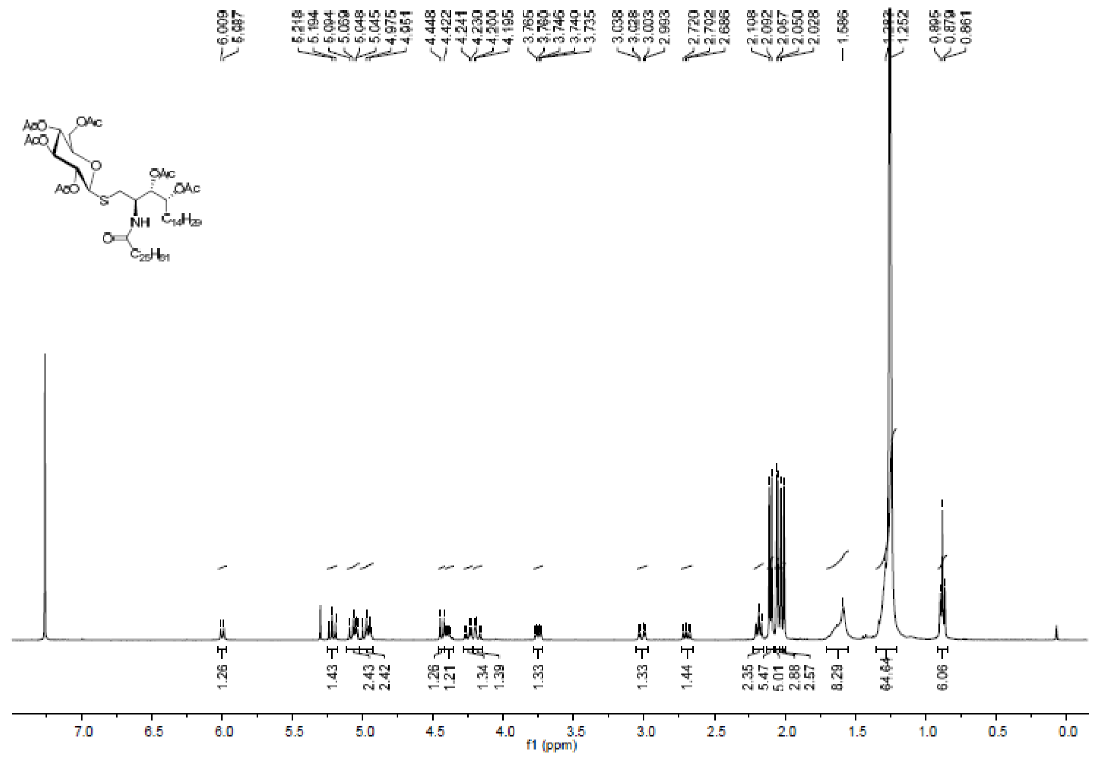

^{1}H-NMR spectrum of (2*R*,3*S*,4*R*)-1-β-D-glucopyranosylthio -2-hexacosanamidooctadecane (**14**, 500 MHz, Pyridine-d5)

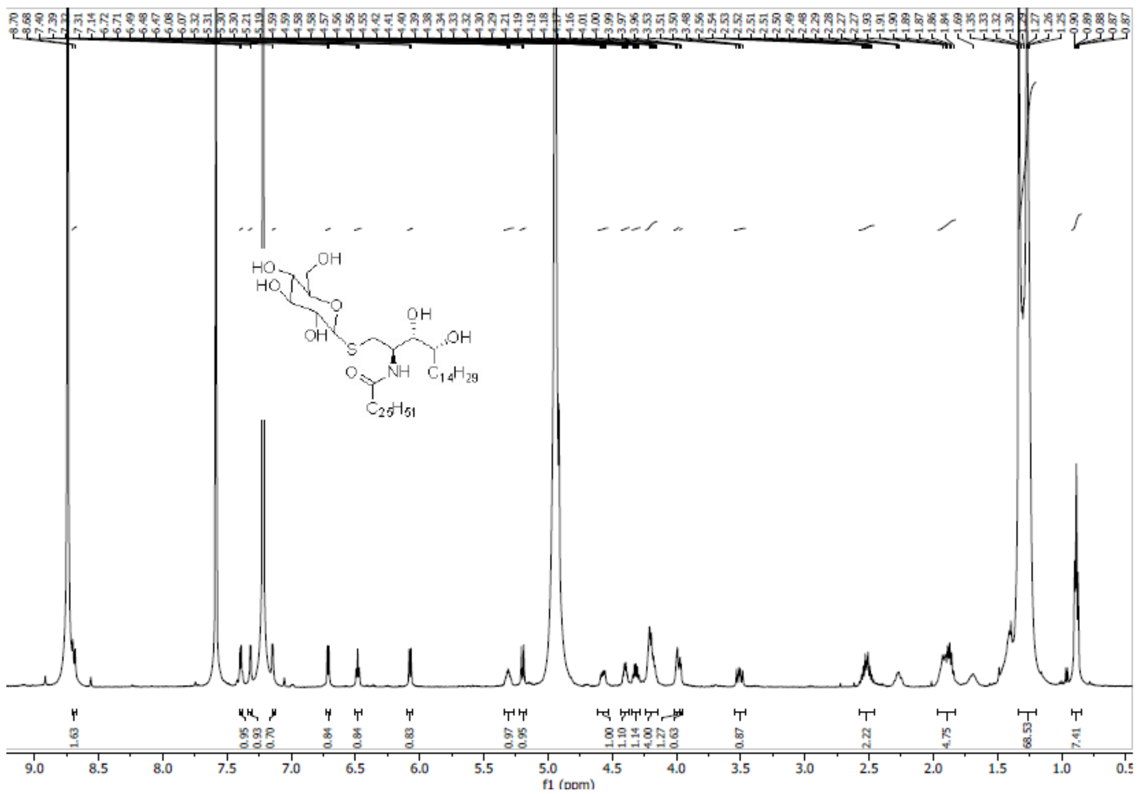

COSY spectrum of (2*R*,3*S*,4*R*)-1-β-D-glucopyranosylthio -2-hexacosanamidooctadecane (**14**, 500 MHz, Pyridine-d5)

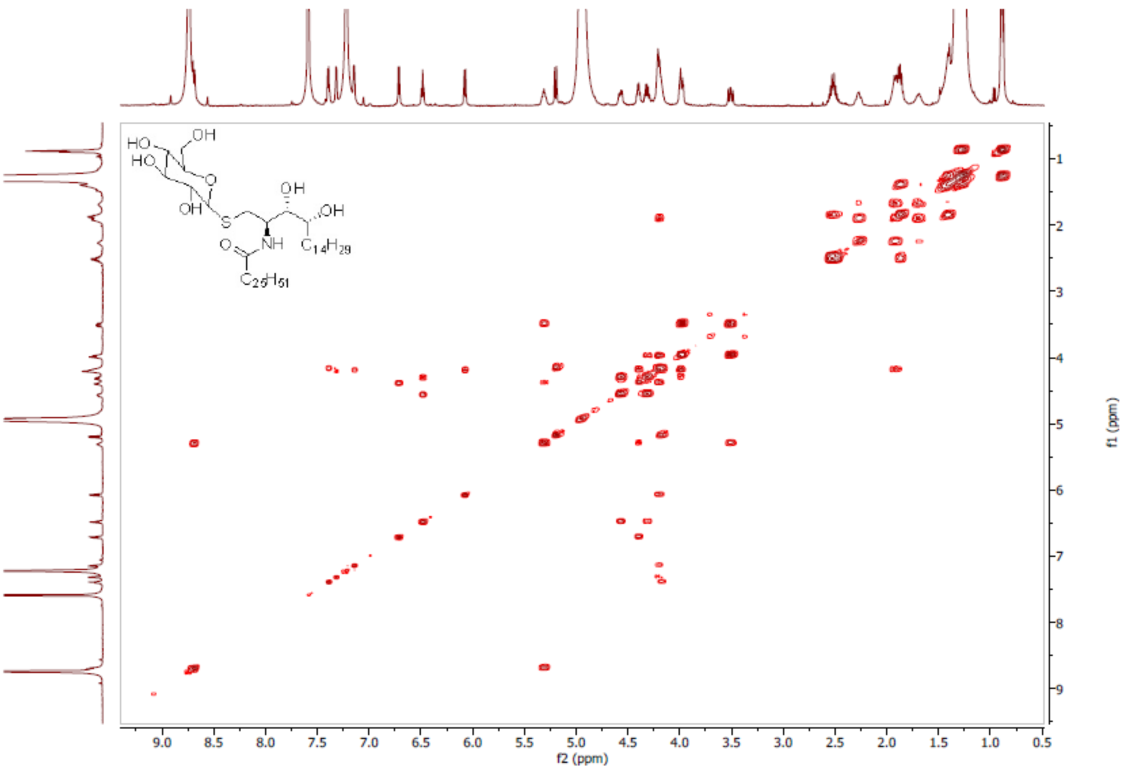

HSQC spectrum of (2*R*,3*S*,4*R*)-1-β-D-glucopyranosylthio -2-hexacosanamidooctadecane (**14**, 500 MHz, Pyridine-d5)

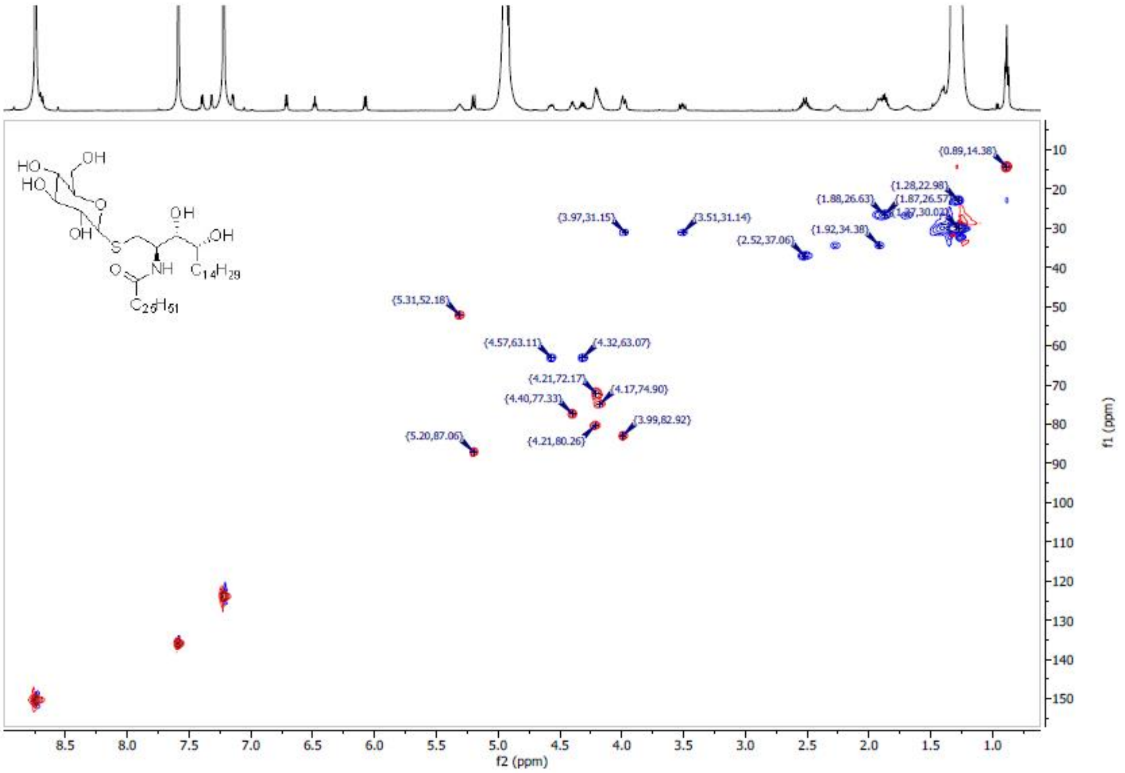

HMBC spectrum of (2*R*,3*S*,4*R*)-1-β-D-glucopyranosylthio -2-hexacosanamidooctadecane (**14**, 500 MHz, Pyridine-d5)

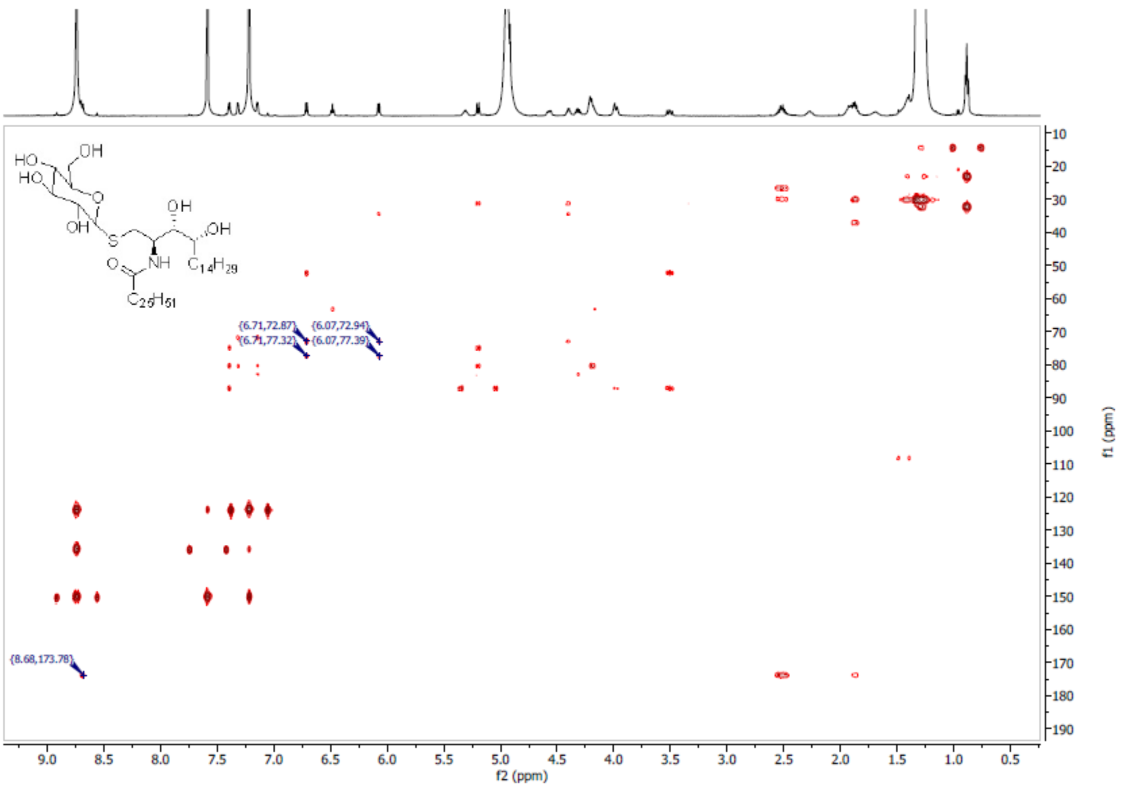

HPLC-ELS of (2*R*,3*S*,4*R*)-1-β-D-glucopyranosylthio -2-hexacosanamidooctadecane (**14**)

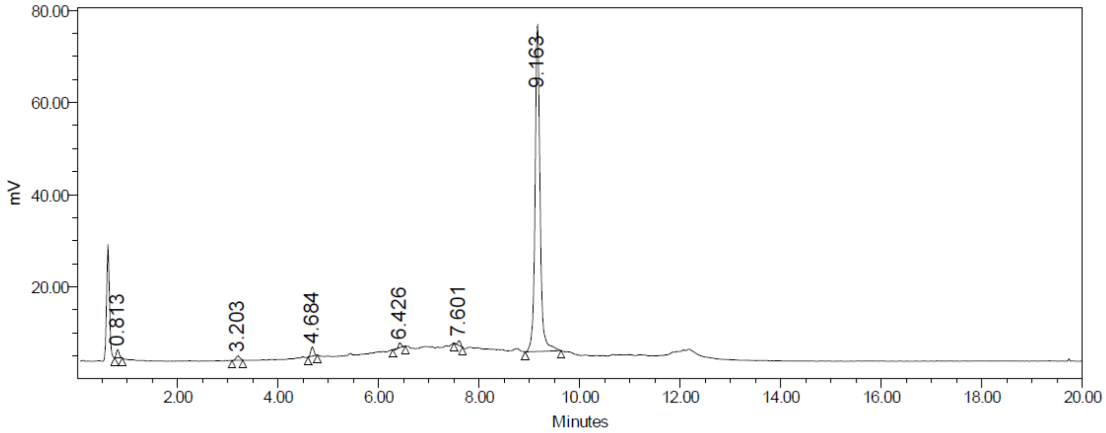

	RT	Area	% Area	Height
1	0.813	6974	1.33	1720
2	3.203	4872	0.93	904
3	4.684	8417	1.61	1966
4	6.426	5029	0.96	1064
5	7.601	4347	0.83	1047
6	9.163	494139	94.34	70589

Bibliography

[1] Y. Harrak, A. Llebaria, A. Delgado, *European J. Org. Chem.* **2008**, *2008*, 4647–4654.

[2] Schrödinger, *Maestro, Version 9.9; Schrödinger, LLC, New York, NY, 2*, **2014**.

[3] J. Kerzerho, E. D. Yu, C. M. Barra, E. Alari-Pahisa, E. Girardi, Y. Harrak, P. Lauzurica, A. Llebaria, D. M. Zajonc, O. Akbari, et al., *J. Immunol.* **2012**, *188*, 2254.

[4] J. López-Sagaseta, J. E. Kung, P. B. Savage, J. Gumperz, E. J. Adams, *PLoS Biol.* **2012**, DOI 10.1371/journal.pbio.1001412.

[5] H. M. Berman, J. Westbrook, Z. Feng, G. Gilliland, T. N. Bhat, H. Weissig, I. N. Shindyalov, P. E. Bourne, *Nucleic Acids Res.* **2000**, *28*, 235–242.

[6] Schrödinger, *Schrödinger Suite 2014 Protein Preparation Wizard; Epik, Schrödinger, LLC, New York, NY, 2014; Impact, Schrödinger, LLC, New York, NY, 2014; Prime, Schrödinger, LLC, New York, NY*, **2014**.

[7] Schrödinger, *LigPrep, Schrödinger, LLC, New York, NY,* **2014**.

[8] Schrödinger, *MacroModel, Version 10.5; Schrödinger, LLC: New York, NY*, **2014**.

[9] L. Banks Jay, S. Beard Hege, Y. Cao, E. Cho Art, W. Damm, R. Farid, K. Felts Anthony, A. Halgren Thomas, T. Mainz Daniel, R. Maple Jon, et al., *J. Comput. Chem.* **2005**, *26*, 1752–1780.

[10] Schrödinger, *Glide, Version 6.4; Schrödinger, LLC, New York, NY*, **2014**.

[11] Schrödinger, *Desmond Molecular Dynamics System, D. E. Shaw Research, New York, NY, 2014. Maestro-Desmond Interoperability Tools, Schrödinger, New York, NY*, **2014**.

[12] D. J. Evans, B. L. Holian, *J. Chem. Phys.* **1985**, *83*, 4069–4074.

[13] G. J. Martyna, M. L. Klein, M. Tuckerman, *J. Chem. Phys.* **1992**, *97*, 2635–2643.

[14] G. J. Martyna, D. J. Tobias, M. L. Klein, *J. Chem. Phys.* **1994**, *101*, 4177–4189.

[15] T. Darden, D. York, L. Pedersen, *J. Chem. Phys.* **1993**, *98*, 10089–10092.

[16] U. Essmann, L. Perera, M. L. Berkowitz, T. Darden, H. Lee, L. G. Pedersen, *J. Chem. Phys.* **1995**, *103*, 8577–8593.

[17] V. Kräutler, W. F. Van Gunsteren, P. H. Hünenberger, *J. Comput. Chem.* **2001**, *22*, 501–508.
